# Cyclooxygenase-2/prostaglandin E2 inhibition remodulated photodynamic therapy-associated immunosuppression for enhanced cancer immunotherapy

**DOI:** 10.1016/j.mtbio.2025.101530

**Published:** 2025-01-28

**Authors:** Tao Xu, Kehan Liu, Shuqi Mi, Yao Yao, Mengyao Zhang, Shujuan Xue, Feng Zhi, Sally-Ann Cryan, Dawei Ding

**Affiliations:** a College of Pharmaceutical Sciences, Soochow University, Suzhou, 215123, China; b School of Pharmacy and Biomolecular Sciences, Royal College of Surgeons in Ireland (RCSI), Dublin, D02 YN77, Ireland; c Department of Gerontology, The Affiliated Suqian Hospital of Xuzhou Medical University, Suqian, 223800, China; d Department of Neurosurgery, The First People's Hospital of Changzhou, Changzhou, 213003, China; e Clinical Medical Research Center, The Third Affiliated Hospital of Soochow University, Changzhou, 213003, China; f Wisdom Lake Academy of Pharmacy, Xi'an Jiaotong-Liverpool University, Suzhou, 215123, China; g Jiangsu Province Higher Education Key Laboratory of Cell Therapy Nanoformulation (Construction), Xi'an Jiaotong-Liverpool University, Suzhou, 215123, China

**Keywords:** Albumin nanoparticles, Photodynamic therapy, Immunogenic cell death, COX-2/PGE2 inhibition, Immunosuppressive tumor microenvironment, PD-L1 blockade

## Abstract

Low immunogenicity and immunosuppressive tumor microenvironment (TME) are two pivotal factors restricting tumor immunotherapy. Photodynamic therapy (PDT) directly destroys cancer cells by producing reactive oxygen species (ROS), and enhances the immunogenicity of "cold" tumors by inducing immunogenic cell death (ICD), thereby promoting T cell development against tumors. However, PDT also deteriorates immunosuppression through overactivating the cyclooxygenase-2/prostaglandin E2 (COX-2/PGE2) pathway. To this end, biocompatible albumin nanoassemblies co-delivering IR780 and diclofenac are herein developed for enhanced therapy against triple-negative breast cancer. PDT-exacerbated PGE2 overexpression is effectively abolished by diclofenac-mediated COX-2 inhibition, which reprograms immunosuppressive TME *via* downregulating the infiltration of various immunosuppressive cells and their cytokine secretion to enhance effector T cell infiltration. Consequently, the enhanced antitumor immunity effectively inhibits tumor growth, prevents the recurrency and metastasis, and remarkably boosts the treatment efficacy of PD-L1 blockade. This study sets an intriguing example for overcoming the COX-2/PGE2 pathway-exacerbated immunosuppression alongside immune activation, thus enhancing synergistic cancer immunotherapy potentiated by various ROS-producing therapies (e.g., PDT and radiotherapy) and chemotherapy.

## Highlights

• An albumin nanoplatform co-delivering IR780 and an NSAID, diclofenac is developed.
• ROS-induced tumor ICD facilitates the development of strong antitumor immunities.
• Diclofenac effectively inhibits PDT-upregulated COX-2 expression and PGE2 secretion.
• COX-2/PGE2 inhibition reverses PDT-related immunosuppression to boost immunotherapy.
• BDIR NPs effectively enhance PD-L1 blockade efficacy *via* promoting CTL infiltration.

## Graphical abstract

**Figure:**
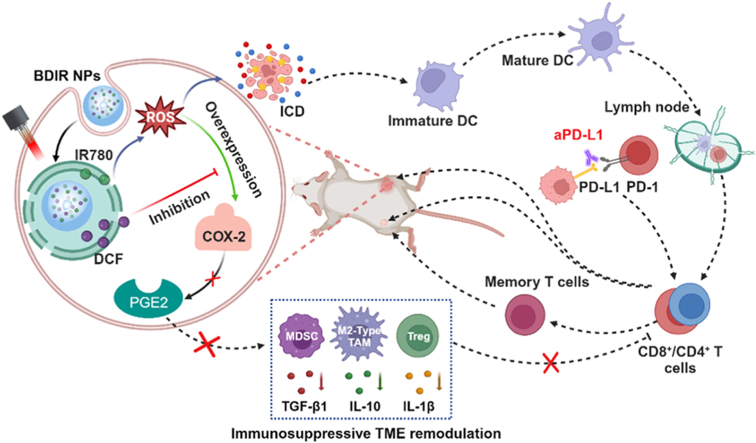

## 1 Introduction

Cancer immunotherapy holds great potential by activating the immune systems to combat malignant tumors. However, treating highly mortal triple-negative breast cancer (TNBC) remains a challenge due to frequent recurrence and metastasis [1]. TNBC's poor immunogenicity and the immunosuppressive tumor microenvironment (TME) represent two critical obstacles of their effective immunotherapy [2]. Immunogenic cell death (ICD) represents a special form of cell death that activates potent anticancer immune responses *via* releasing tumor antigens and producing various damage-associated molecular patterns (DAMPs) like calreticulin (CRT), adenosine triphosphate (ATP) and high mobility group protein 1 (HMGB1) [3]. These danger signals promote dendritic cells (DCs) maturation, thereby stimulating innate and adaptive immunity [4,5]. ICD can be induced in cancer cells through various therapeutic modalities like chemo-, radio-, or photo-therapy [3]. In particular, photodynamic therapy (PDT) is a noninvasive and selective modality that utilizes appropriate wavelength of light to excite a photosensitizer (PS) and produce cytotoxic reactive oxygen species (ROS) for direct tumor ablation and induction of systemic anti-tumor immunities [[6], [7], [8], [9]].

Despite the immune stimulation, research evidences also suggest that PDT can paradoxically trigger harmful protumorgenic effects, such as the overproduction of cyclooxygenase-2 (COX-2) and the resultant secretion of prostaglandin E2 (PGE2) in a broad spectrum of tumors such as colorectal, gastric, esophageal, pancreas, lung and breast cancers [[10], [11], [12], [13], [14], [15], [16]]. The inflammatory COX-2/PGE2 pathway contributes to the formation, progression, and metastasis of cancers by influencing the angiogenesis, cancer cell proliferation and suppression of anti-tumor immunity [16,17]. As an immunosuppressor, PGE2 could facilitate tumor growth by impeding DCs function, enhancing regulatory T cell (Treg) activation, and inhibiting the cytotoxic T lymphocyte (CTL) expansion through PGE2-EP2/EP4 signaling [18,19]. PGE2 is also associated with the myeloid-derived suppressor cells (MDSC)-mediated immunosuppression and polarization of tumor-associated macrophages (TAMs) from an anti-tumoral M1 to an immunosuppressive M2 phenotype, thus collectively suppressing the activity of CTLs [20,21]. Moreover, tumor-derived PGE2 is also reported to lower tumors’ response to immune checkpoint blockade (ICB) [22].

**Scheme 1:**
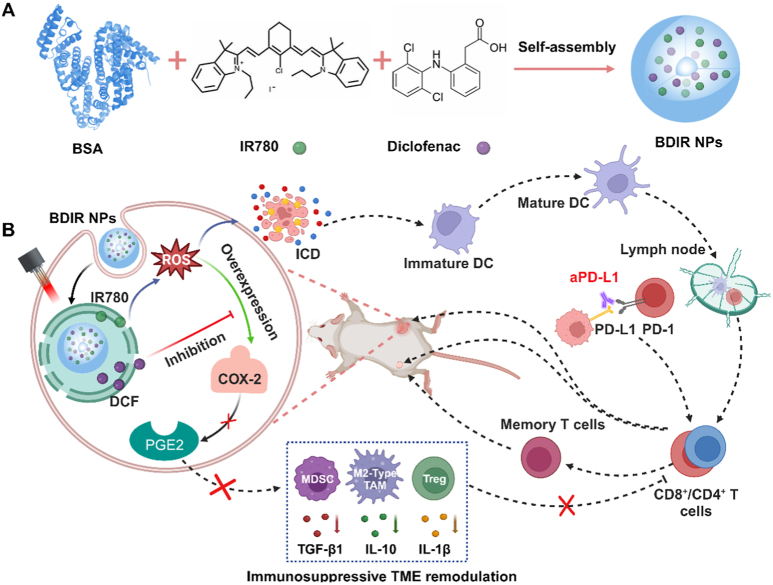
Schematic overview of BDIR NPs for the combination of PDT and COX-2/PGE2 pathway inhibition against TNBC. (A) The preparation process of BDIR NPs. (B) Schematic elucidation of the mechanism of improved immunotherapy of BDIR NPs by simultaneous ICD elicitation and COX-2/PGE2 inhibition which enhances the immunogenicity and reverses the immunosuppressive microenvironment at the same time.

Considering the negative role of COX-2/PGE2 in forming immunosuppressive TME and the exacerbation of COX-2 expression by PDT, the abolishment of PGE2 production rises as a viable strategy for enhancing PDT-potentiated tumor immunotherapy. Clinical and epidemiologic evidence have shown that regular use of nonsteroidal anti-inflammatory drugs (NSAIDs) confers chemoprevention against breast cancer formation, as NSAIDs inhibit COX-2-catalyzed PGE2 production [[23], [24], [25]]. Given this, we herein present a bovine serum albumin (BSA)-based nanoplatform co-delivering IR-780 iodide (IR780) as the photosensitizer and an NSAID, diclofenac (DCF) as the COX-2 inhibitor [26] (referred as BDIR NPs) to maximize the efficacy of PDT-powered immunotherapy for TNBC. Upon tumor accumulation and near-infrared light (NIR) irradiation, IR780 generates ROS and induces ICD, which promotes the development of tumor-specific CTLs. Meanwhile, DCF inhibits COX-2 and reverses immunosuppressive TME by decreasing PGE2 level, thus suppressing the infiltration of various major immunosuppressive cells and promoting the filtration and efficacy of CTLs. The boosted anti-tumor immunity not only ablates primary tumors, but also effectively suppresses distant and re-challenged tumors *via* abscopal and immune memory effects, and effectively boosts the efficacy of ICB by PD-L1 monoclonal antibodies (aPD-L1), especially achieving a remarkable suppression of distant tumors (Scheme 1). This study explores a novel immunotherapeutic strategy that combines PDT and COX-2/PGE2 inhibition to reshape the deteriorated immunosuppressive TME, tackling the negative side of “double-edged sword” for PDT which has been broadly overlooked in ROS-powered immunotherapy of cancers. Moreover, given the upregulated COX-2 expression widely caused by chemotherapy and other ROS-producing therapies (e.g., radiotherapy) [13,27], it also offers constructive insights to enhance the synergistic immunotherapy potentiated by these clinically applicable treatments.

## 2 Materials and methods

### 2.1 BDIR NPs preparation and characterization

IR780 and diclofenac (DCF) were loaded into BSA through a self-assembly method [28]. In detail, 100 mg of BSA powder was dissolved in 18 mL PBS (pH7.2 10 mM) with constant stirring at 37 °C in a water bath for 0.5 h. Then a mixture of ethanol solutions containing IR780 (2 mg/mL in stock solution) and DCF (20 mg/mL in stock solution) was slowly added to the BSA solution and stirred away from light for 2 h. Afterwards, the self-assembled nanoparticles were cooled down, and filtered to remove any possible large aggregates. Then the mixture was purified using ultrafiltration tubes (100 kDa, Millipore) through about 5 cycles of ultrafiltration process (1500 rcf/min, 5 min, 4 °C) to remove unencapsulated free drugs and unassembled BSA molecules. Subsequently, the obtained IR780 and DCF-co-loaded BSA nanoparticles (referred as BDIR NPs) were filtrated through 0.22 μm PFS syringe filters. The same process was conducted to prepare IR780-loaded BSA nanoparticles (termed as BIR NPs) and diclofenac-loaded BSA nanoparticles (termed as BD NPs) as controls. The Fourier-transform infrared (FTIR) characterization, drug loading content, encapsulation efficiency, colloidal and photo-stability, as well as drug release of BDIR NPs were assessed with details provided in the Supplementary Information.

To evaluate the PDT properties of BDIR NPs, singlet oxygen sensor green (SOSG) was used to examine singlet oxygen (^1^O_2_) generation in IR780 formulations by detecting the fluorescence intensity at 525 nm using a multifunctional microplate reader (Tecan Infinite M1000 PRO, USA). Briefly, 200 μL different samples (BDIR NPs, BIR NPs, free IR780 at 1 μg/mL IR780) and indocyanine green (ICG) standard control were added into a black 96-well plate. Then 20 μL of SOSG (50 μM) was mixed with the samples, and the initial fluorescence intensity was recorded. Each well was exposed to NIR irradiation (808 nm, 0.5 W/cm^2^) and the oxidized SOSG was measured by recording the fluorescence intensity every 5 s for ^1^O_2_ calculation or every 60 s for the ROS generation plot. The fluorescence of each sample without SOSG at 808 nm were also recorded. All operations were performed in triplicate in the dark room. The singlet oxygen quantum yield (Φ_Δ_) was calculated by following equation [29]:

$$\Phi_{\Delta }=\Phi_{\Delta \text{ICG}}\times \left(\gamma /A\right)\;/\;\left(\gamma_{\text{ICG}}/A_{\text{ICG}}\right)$$

where γ and γ_ICG_ represent the reaction rates of the SOSG with ^1^O_2_ produced by IR780 and ICG, respectively. A and A_ICG_ are the absorbances of IR780 and ICG at 808 nm.

### 2.2 Cytotoxicity and cellular apoptosis

Cytotoxicity of BDIR NPs with or without exposure to light was tested using CCK-8 assay. In brief, 4T1 cells (6 × 10^3^ cells/well) were plated into a 96-well plate and left to grow overnight. Afterwards, the culture medium was exchanged for fresh medium containing various formulations (PBS, BD NPs, BIR NPs, BIR NPs + L, BDIR NPs and BDIR NPs + L) at a range of concentrations of IR780 (1–20 μM) or DCF (22–451 μM) and continued with the incubation for 24 h. Subsequently, the cells were rinsed, refreshed with new medium and exposed to NIR irradiation (808 nm laser, 0.5 W/cm^2^, 5 min) for the “+L” groups (same as below for *in vitro* studies) and continuously incubated for 24 h. CCK-8 was added to determine cell viability following standard instructions. In addition, cell apoptotic level was analyzed, as detailed in the Supplementary Information.

### 2.3 Intracellular ROS generation

The ROS production in tumor cells was assessed by the 2ʹ,7ʹ-dichlorodihydrofluorescein diacetate (DCFH-DA) assay analyzed by confocal laser scanning microscopy (CLSM) and flow cytometer (FC). For CLSM analysis, 2 × 10^5^ cells 4T1 cells were plated into a confocal dish and left for attachment. Cells were then treated with PBS, BD NPs, BIR NPs, BIR NPs + L, BDIR NPs and BDIR NPs + L (2 μg/mL IR780, 20 μg/mL DCF) as above. 6 h post-treatment, the DCFH-DA solution in PBS was added and incubated in dark for 30 min. Subsequently, the cells in the “+L” groups were subjected to NIR irradiation (5 min, 0.5 W/cm^2^) and observed by CLSM after the incubation with DAPI (5 μg/mL) for nuclear staining. For the quantitative determination of ROS by flow cytometry, a similar protocol as described above was used, except that the cells after staining were collected by trypsin digestion for FC instead of CLSM.

### 2.4 COX-2/PGE2 inhibition *in vitro*

To verify the intracellular DCF-related inhibition of COX-2 and reduction of PGE2 level, 4T1 cancer cells (1 × 10^5^ cells/well) were distributed into 24-well plates and incubated overnight. Then the cells were treated with PBS, BDIR NPs, BIR NPs, BIR NPs + L, BDIR NPs and BD NPs + L as above. Afterwards, the medium supernatants (1 mL) were harvested and centrifugated to remove cell debris. The resulting supernatant was then used for quantifying PGE2 levels *via* a high sensitivity competitive enzyme-linked immunosorbent assay (ELISA) method [30]. Total proteins from the treated 4T1 cells were extracted and quantified for the measurements of COX-2 expression. The samples (50 μg each) were separated by 10 % gel electrophoresis, then transferred onto polyvinylidene fluoride (PVDF) membrane, and further incubated with COX-2 antibodies at 4 °C overnight. Afterwards, the membrane was treated with a secondary antibody for 1 h at room temperature and visualized with ECL plus detection system (CHEMIDOC, Bio-Rad, USA).

### 2.5 ICD *in vitro*

To investigate CRT surface expression, HMGB1 release, and ATP secretion *in vitro*, 1 × 10^5^ cells 4T1 cells were placed into each well of a 24-well glass-bottom plate and incubated overnight. They were then treated with PBS, BDIR NPs, BIR NPs, BIR NPs + L, BDIR NPs and BD NPs + L as above (2 μg/mL IR780, 20 μg/mL DCF) for 24 h. Six hours post the NIR irradiation for “+L” groups, the cells were then fixed, blocked, and incubated with anti-CRT primary antibodies (1:200) or HMGB1 primary antibodies (1:200) at 37 °C for 3 h or 1.5 h, respectively. After washing with TBST, the cells were incubated in the dark with FITC-labeled secondary antibodies (1:200) for an additional 1 h. They were stained with DAPI before the observation by CLSM. Furthermore, the cell supernatants were collected through centrifugation (400 *g*, 5 min) for the assay of ATP level with ATP assay kit following the supplier's guidelines.

### 2.6 DC maturation *in vitro*

Murine dendritic cell line DC2.4 was used to study dendritic cell maturation and activation. The process involved seeding 4T1 cells in 6-well plates and subjecting them to various treatments as described above. Afterwards, the culture medium was collected, centrifuged (1000 *g*, 10 min) to remove any cellular debris, and used as tumor-conditioned media to culture DCs. Twenty-four hours later, DCs were collected and resuspended in staining buffer solution (PBS with 1 % FBS and 2 mM EDTA). The cells were then blocked with Fc receptor blocking reagent for 5 min at 4 °C. In the following, they were stained with 100 μL of staining solution containing anti-CD80-APC and anti-CD86-PE in dark at 4 °C for 30 min before washing and flow cytometry analysis.

### 2.7 Murine carcinoma models

All conducted animal studies were reviewed and approved by the Ethics Committee of Soochow University (China). Female Balb/c mice (6–8 weeks, 16–18 g) were obtained from the Beijing Vital River Laboratory Animal Technology Co., Ltd. and settled-down in a specific pathogen-free (SPF) laboratory for 1 week. For single tumor model, 1 × 10^6^ 4T1 cells suspended in 50 μL of sterile PBS were injected subcutaneously (*s.c.*) into the right flank of each mouse. In the bilateral tumor model, distant tumors were similarly developed on the other side with 4T1 cells after the primary tumors grew to ∼100 mm^3^. The tumor volume (V) was measured based on the formula:

V=(Length×Width2)2

### 2.8 Biodistribution *in vivo*

To assess the tissue distribution of BDIR NPs, single subcutaneous 4T1 tumor mice models were established. When the tumor size reached the desired volume (150–200 mm^3^), mice were grouped (2 group, n = 3) and intravenously (*i.v.*) injected with BDIR NPs and free IR780, respectively with equivalent dose of IR780 (100 μL, 2 mg/kg). To facilitate imaging, the mice were anesthetized in a chamber flowed with 3 % isoflurane in oxygen atmosphere and imaged *via* an *in vivo* imaging system (IVIS) (IVIS Lumina, USA) at scheduled time points of 0, 3, 6, 12, 24, 48 and 72 h post-injection. 72 h post-imaging, the mice were then sacrificed by euthanasia, while key organs including heart, liver, spleen, lung, kidney and tumor were harvested to analyze the tissue distribution of BDIR NPs through fluorescence intensity measurements.

### 2.9 Anti-tumor efficacy in bilateral tumor model

To study the anti-tumor effect in the bilateral tumor model, six groups of tumors-implanted mice (n = 5) were randomly selected and treated with PBS, BD NPs, BIR NPs, BIR NPs + L, BDIR NPs and BDIR NPs + L (2 mg/kg IR780, 20 mg/kg DCF) every three days (3 times in total). When the primary tumor size reached 120∼150 mm^3^, NIR irradiation (808 nm, 1 W/cm^2^, 5 min) was only applicable to primary tumors of “+L” groups (same as below for *in vivo* studies). A higher laser power (1 W/cm^2^) is applied to ensure adequate light penetration and photosensitizer activation at the target site *in vivo* [9,31,32]. Tumor volume and mice weight were measured every other day until day 28. The survival rate was recorded throughout the experiment. Mice were euthanized upon tumor volume exceeded 1500 mm^3^ or tumors became necrotic.

### 2.10 Intratumoral infiltrations of immune cells and secretion of cytokines

To investigate the DCs maturation, intratumoral infiltration of T cells, MDSCs and TAMs, mice with bilateral 4T1 tumors were allocated into 6 groups (n = 3) and received diverse formulation/NIR irradiation for 3 rounds as above. After seven days of the treatment regimen, mice were euthanized to harvest primary and distant tumors for analysis of CTLs, MDSCs, Tregs and TAMs, as well as collect tumor-draining lymph nodes (TDLNs) for matured DCs assessment. For analyzing DC condition in the TDLNs, the lymph nodes were ground by syringe pistols to prepare single-cell suspension, which was then stained with mixture of anti-CD11c-FITC, anti-CD80-PE, anti-CD86-APC for DC maturation detection (CD80^+^CD86^+^). For analyzing immune cell populations in the tumor tissues, single-cell suspensions were prepared using enzymatic digestion, grinding, and filtering of tumors. The filtered single cell suspensions were then stained with a panel of antibodies: CTLs (e.g., anti-CD3-Percp/Cy5.5, anti-CD8a-PE, anti-mouse CD4-FITC), Tregs (anti-CD3-Percp/Cy5.5, anti-CD4-FITC, anti-CD25-APC, anti-FOXP3-PE), MDSCs (anti-CD45-PE, anti-CD11b-FITC, anti-Gr-1-APC), and TAMs (anti-CD11b-FITC, anti-F4/80-APC, anti-CD86-PE/Cyanine7, anti-CD206-PE). After staining, the samples were analyzed using BD ARAIII flow cytometer. In parallel, another set of six groups (n = 3) with the same treatments were used for cytokine analysis. After tumor excision, washing, and homogenization, the homogenates were centrifuged to separate supernatants, which were then analyzed using ELISA kits to examine the levels of TNF-*α*, IFN-*γ*, TGF-β1, IL-1β and IL-10, following the manufacturers’ protocols.

### 2.11 Tumor rechallenge and prevention of metastasis

To investigate the long-term immune memory and metastasis resistance resulting from the combinatorial therapy, 4T1 cells were injected (*s.c.*, 1 × 10^6^ cells/mouse) into the right hind flank of each mouse to initiate the primary tumor. After the primary tumor volume reached 50–60 mm^3^, they were either surgically resected or treated three times with BDIR NPs + L (n = 7). After 30 days, the rechallenged 4T1 tumors were inoculated (*s.c.*, 5 × 10^5^ cells/mouse) in the contralateral side. The growth of rechallenged tumors was monitored during experimental time. For metastasis assessment, the lungs and livers from both deceased mice and those euthanized at the experiment's end were harvested. These tissues were photographed, fixed, and processed for histopathology analysis (H&E staining). In a separate experiment, 3 mice in each group received the same treatments on primary tumors were euthanized 1 day before secondary tumor rechallenge. The spleens were isolated, and FC was used to determine the percentages of effector T memory cells (T_em_: CD8^+^CD44^+^CD62L^−^) and central T memory cells (T_cm_: CD8^+^CD44^+^CD62L^+^). Additionally, serum samples collected from mice 7 days after rechallenging were analyzed using ELISA kits to quantify TNF-α and IFN-γ level.

### 2.12 Anti-tumor effect in combination with anti-PD-L1 antibody (aPD-L1)

In the bilateral 4T1 tumor model, mice were allocated to 3 groups and treated with PBS, aPD-L1 (2 mg/kg, *i.p.*) and BDIR NPs + L + aPD-L1 (2 mg/kg IR780, *i.v.*; 2 mg/kg aPD-L1, *i.p.*). aPD-L1 was administered after NIR irradiation (3 times in total). Tumor volume and mice weight were checked every two days. Mice survival rates were monitored throughout the experiment. To assess the tumor-infiltrating immune T cells, 3 mice in each group were euthanized for preparing tumor single cell suspensions, which were then stained with the mixture antibodies of anti-CD3-Percp/Cy5.5, anti-CD8a-PE and anti-mouse CD4-FITC, and analyzed using a BD ARAIII flow cytometer.

### 2.13 Statistical analysis

Data were expressed as means ± standard deviation (SD). One-way analysis of variance (ANOVA) and Student's t-test were used to determine statistical significance. ∗∗∗*p* < 0.001, ∗∗*p* < 0.01 and ∗*p* < 0.05 were considered statistically significant. ns indicates no significance.

## 3 Results and discussion

### 3.1 Preparation and characterization of BDIR NPs

**Fig. 1:**
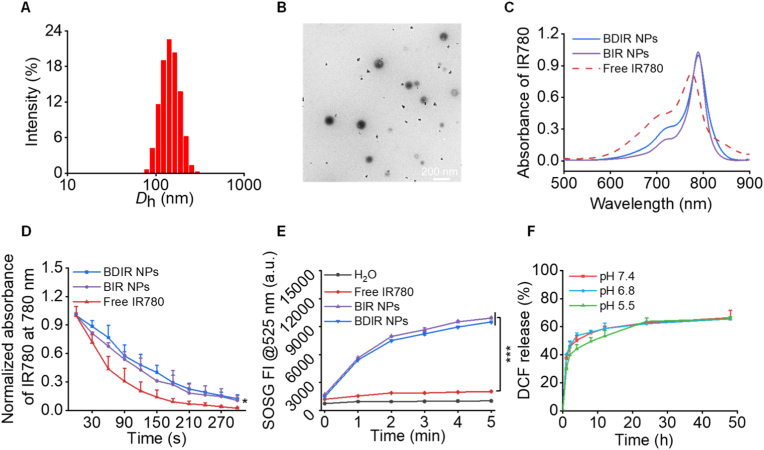
**Characterization of BDIR NPs**. (A) Hydrodynamic diameter (*D*_h_) of BDIR NPs analyzed by DLS. (B) TEM images of BDIR NPs. Scale bar = 200 nm. (C) UV–vis spectra of IR780 in different formulations. (D) Photostability of various samples measured by UV–Vis absorbance. (E) ROS generation of BDIR NPs revealed by SOSG under NIR irradiation (808 nm laser, 0.5 W/cm^2^). (F) Release profile of DCF from BDIR NPs at different pH conditions. Results are expressed as the mean ± standard deviation (SD), n = 3, statistical significance was determined by Student's t-test, ∗*p* < 0.05, ∗∗∗*p* < 0.001.

BSA was employed as a nanocarrier to fabricate nanoparticles co-delivering IR780 and DCF (referred as BDIR NPs) *via* hydrophobic interactions-driven self-assembly under a mild condition (Scheme 1A) [9]. The optimized BDIR NPs exhibited a hydrodynamic diameter (*D*_h_) of 121.6 ± 5.4 nm with a narrow distribution (PDI: 0.157 ± 0.012) (Fig. 1A), slightly larger than the corresponding single drugs-loaded formulations, e.g., 97.6 nm for IR780-loaded nanoparticles (BIR NPs) and 106.9 nm for DCF-loaded nanoparticles (BD NPs) (Figs. S1A and B, Table S1). Furthermore, BDIR NPs retained excellent colloidal stability over a week (Fig. S1C). This may be attributed to their zeta potential of −18.3 ± 0.6 mV at pH 7.4 (Table S1), which indicated an efficient electrostatic repulsion between NPs in the aqueous dispersion [33]. Besides, the negative charge tends to offer better *in vivo* biodistribution by reducing immune cell uptake (especially by macrophages) and toxicity [34]. TEM image revealed a spherical morphology with an average size around 87.0 ± 19.7 nm for BDIR NPs due to dehydration (Fig. 1B). BD NPs and BIR NPs also displayed generally smaller dimensions by TEM observations than DLS (Figs. S1D and E, Table S1). These dimensions could endow the NPs with enhanced EPR effect *in vivo*, which was crucial for tumor accumulation [35]. To further confirm the formation of BDIR NPs, the FTIR spectra of drugs, BSA and lyophilized BDIR NPs were analyzed (Fig. S1F). The characteristic peaks assigned to IR780 (C=C bonds at 1551, 1515, 1366 cm^−1^; C-H bond at 1250 cm^−1^) [36] and DCF (C-Cl bond at 740 cm^−1^; C-H bond at 1272 cm^−1^; C=C bond at 1690 cm^−1^; N-H bond at 3322 cm^−1^) [37] were observed. The main peaks of BSA included 3286 (N-H), 2957 (N-H^3+^), 1645 (C=O in amide I), 1519 (C-N and N-H in amide II), 1389 (C-H_2_) and 1082 cm^−1^ (C-N and N-H in amide III) [38]. The shift of the characteristic peak positions of BSA (C=O from 1645 cm^−1^ to 1650 cm^−1^; C-N and N-H from 1519 cm^−1^ to 1527 cm^−1^; C-H_2_ from 1389 cm^−1^ to 1396 cm^−1^) as well as the presence of 1154, 1096 and 797 cm^−1^, likely due to the hydrophobic interaction between BSA and the drugs [[39], [40], [41]], confirmed the successful formation of the drug-loaded BDIR NPs. The drug loading content of IR780 and DCF in BDIR NPs was about 1.1 ± 0.2 % and 11.9 ± 1.5 % by UV–vis and HPLC analysis, respectively (Table S1).

The UV–vis absorbance spectra of BDIR and BIR NPs revealed comparable profiles with a red shift of absorbance to 789 nm by BSA encapsulation compared to free IR780 (Fig. 1C), which was in accordance with our recent study and might be attributed to hydrophobic interactions between IR780 and BSA [9]. Reasonably, the increase of water solubility for IR780 and decrease of aggregation by BSA encapsulation accounted for an improved photostability (Fig. 1D), increased singlet oxygen generation and the quantum yield (Fig. 1E & S1G) of BDIR NPs, both of which are favorable for PDT. Meanwhile, IR780 revealed negligible release (<5 %) within 24 h (Fig. S1H), which was beneficial for ROS generation in the encapsulated state. In contrast, DCF showcased a much faster release pattern, reaching about 60 % within 24 h at different pH conditions (Fig. 1F), ensuring a more gradual and sustained release for potential immune re-modulation. We speculate that the similarity in DCF release across the three pH conditions could be attributed to a few factors. Firstly, unlike other pH-sensitive drug delivery systems, BSA NPs may maintain their structural integrity and functionality across a broad pH spectrum due to the outstanding stability of BSA [42]. This means that BSA NPs might not undergo significant changes in their physicochemical properties, such as size, surface charge, or porosity, which could influence drug release. Secondly, DCF release from BSA NPs might be governed more by diffusion mechanism that is less dependent on pH [43]. If the release is controlled by a non-pH-sensitive mechanism, there may be minimal changes in release profiles across the different pH conditions. Thirdly, as a weak acid with a pKa of 4 [44], diclofenac may still be sufficiently soluble at all three pH conditions (7.4, 6.8 and 5.5) beyond the pKa to allow its release from the nanoparticle. This could also be explained by the slightly slower release at pH 5.5 from 2 to 24 h (Fig. 1F), but such difference was not enough to influence the entire release. These findings highlight the role of BSA encapsulation in enhancing the performance of both therapeutic agents.

### 3.2 Cellular uptake, cytotoxicity, apoptosis and ROS generation of 4T1 cells

**Fig. 2:**
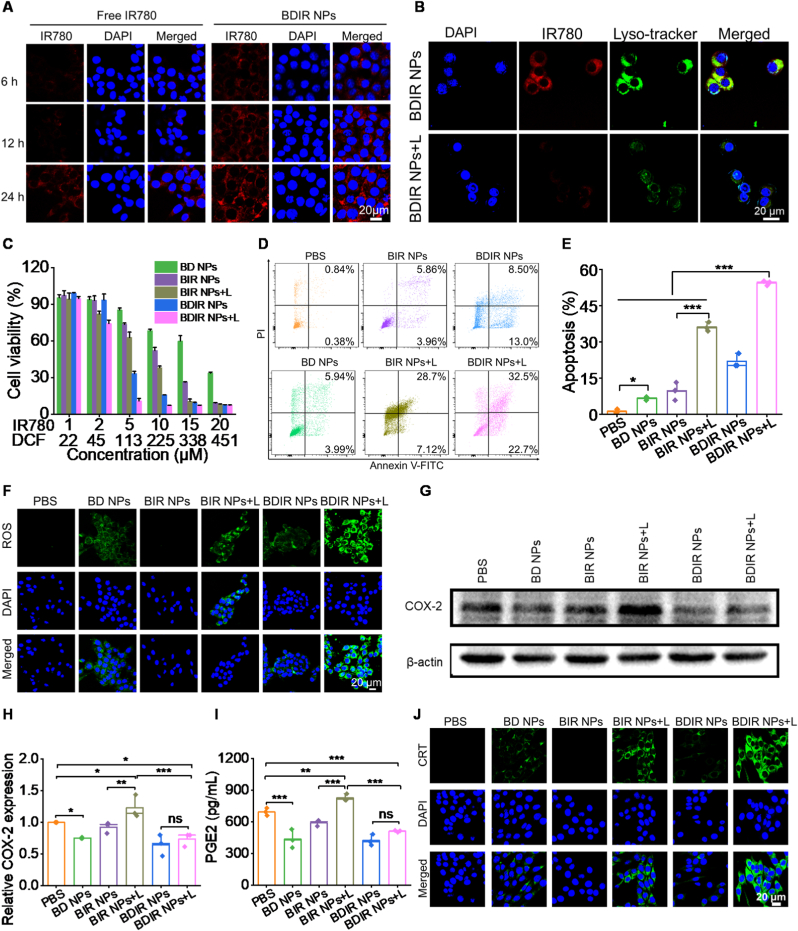
***In vitro* study of BDIR NPs.** (A) Confocal images showing the cellular uptake of free IR780 and BDIR NPs in 4T1 cells. Scale bar = 20 μm. (B) Co-localization of BDIR NPs with lysosome in 4T1 cells and the lysosomal escape upon NIR irradiation. Scale bar = 20 μm. (C) Relative cell viability of 4T1 cells treated with different NPs for 24 h at various concentrations with or without NIR irradiation (808 nm, 0.5 W/cm^2^, 5 min) (n = 3). (D) Flow cytometry plots showing representative 4T1 cell apoptosis under various treatments. (E) Quantification of cell apoptosis percentage (n = 3). (F) Confocal microscopic analysis of intracellular ROS generation. Scale bar = 20 μm. (G) Western blot analysis of COX-2 expression in 4T1 cells exposed to various treatments and (H) densitometric analysis of COX-2 expression normalized to β-actin as the loading control (n = 3). (I) PGE2 levels measured in the cell supernatants of 4T1 after treatments with various formulations (n = 3). (J) Confocal microscopic images of CRT exposure of 4T1 cells treated with different formulations. Scale bar = 20 μm. Results are expressed as the mean ± standard deviation (SD), statistical significance was determined by one-way ANOVA, ∗*p* < 0.05, ∗∗*p* < 0.01, ∗∗∗*p* < 0.001, ns: no significance.

CLSM images revealed that BDIR NPs displayed a time-dependent uptake and accumulated in 4T1 cells faster than free IR780, while the flow cytometry analysis confirmed a nearly doubled fluorescence intensity by BDIR NPs (Fig. 2A & S2). Subcellular distribution indicated a co-localization of BDIR NPs with lysosomes (yellow fluorescence due to the overlap of red and green ones) with a Pearson's correlation coefficient of 0.8 (Fig. 2B & S3). Moreover, the green fluorescence obviously faded after NIR irradiation, which manifested that PDT could promote cytoplasmic distribution of NPs *via* lysosome disruption and benefit the following drug release, being particularly meaningful for DCF's activity. The CCK-8 assay indicated that BD NPs treatment for 24 h did not cause significant cell death, as shown by a cell viability exceeding 80 % even at a concentration over 200 μM (Fig. 2C). The cell viabilities in other groups showed a concentration-dependent decrease. Particularly, the combination of NIR irradiation with BIR NPs exhibited substantial suppression of cell growth with an IC_50_ of 5.93 μM for IR780, whereas BDIR NPs under irradiation reduced the IC_50_ to 3.36 μM (Fig. S4), accentuating the synergistic potency of IR780 and DCF. Additionally, BDIR NPs triggered the highest apoptotic level at 54.5 % under NIR irradiation, compared to 21.9 % without NIR irradiation (Fig. 2D and E). Besides the total apoptotic level, the proportion of late apoptotic/necrotic cells based on Annexin V-FITC^+^/PI^+^ was also plotted (Fig. S5A). The data revealed that BDIR NPs + L treatment led to significantly higher late apoptosis/necrosis (32.5 %) compared to other groups including BIR NPs + L treatment (Fig. S5B). This was likely due to both effective endocytosis and photo-activatable ROS generation. The intracellular ROS generation was examined by DCFH-DA probe [45]. CLSM images clearly revealed abundant ROS production triggered by NIR irradiation in all groups containing IR780 compared to PBS control and non-irradiated NPs (Fig. 2F). BD NPs group without NIR irradiation also displayed increased fluorescence, properly related to mitochondrial dysfunction caused by DCF [46]. Reasonably, BDIR + L treatment led to the brightest green fluorescence.

### 3.3 COX-2/PGE2 pathway inhibition *in vitro*

To validate the intracellular COX-2/PGE2 pathway regulation, COX-2 expression and PGE2 secretion were measured *via* western blot and ELISA, respectively. Compared to PBS, BIR NPs without NIR irradiation caused no difference in COX-2 expression, while BIR NPs under irradiation increased that by ∼33 % (Fig. 2G and H). This suggested the COX-2/PGE2 pathway upregulation by PDT, as reported in previous study [47]. To the contrary, BDIR NPs showed significant decrease of COX-2 expression with irradiation compared to either PBS or BIR NPs + L, suggesting the powerful suppression of COX-2 production by DCF. Similarly, compared to the PBS control, cells exposed to BIR NPs and NIR irradiation released significant quantities of PGE2 (829.0 ± 33.9 pg/mL) (Fig. 2I), staying in agreement with the COX-2 expression. In contrast, BD NPs led to significantly decreased secretion of PGE2 (438.2 ± 88.4 pg/mL). Cells treated with BDIR NPs + L were also significantly decreased PGE2 secretion compared with PBS group, which was even comparable to BDIR NPs without NIR irradiation. Overall, these data demonstrated that reduced PGE2 production was directly correlated with COX-2 inhibition, suggesting DCF-based COX-2/PGE2 suppression holds the potential to benefit PDT-induced immunotherapy.

### 3.4 ICD and DC maturation *in vitro*

The ability of BDIR NPs to induce ICD cascade in 4T1 cells was evaluated by measuring the specific molecular events including CRT surface exposure, HMGB1 release, and ATP secretion. CRT is recognized as the primary biomarker of ICD, as it signals antigen-presenting cells, particularly DCs, to phagocytose the dying cells [48]. HMGB1, another ICD biomarker acting as a Toll-like receptor agonist, facilitates antigen presentation and DC maturation [49]. The 4T1 cells treated with laser-activated BIR NPs displayed apparent CRT exposure according to the intense green fluorescence (Fig. 2J), indicating effective ICD induction by PDT. Interestingly, BD NPs also triggered mild CRT exposure, possibly attributed to the DCF-mediated mild ROS production. To our knowledge, there are few reports of DCF's role in inducing tumor ICD. Consequently, the highest CRT expression was observed in BDIR NPs + L-treated cells (Fig. S6A), suggesting that BDIR NPs could induce more noticeable ICD. Similarly, cells treated with BDIR NPs + L showed the most significant release of HMGB1 from cell nuclei (Figs. S6B and C) and much increased ATP secretion (Fig. S6D) compared to all other groups. Collectively, these results implied that BDIR NPs could efficiently enter tumor cells and elicit powerful ICD through the synergy of PDT and DCF. Enhanced ICD contributed to the highest proportion of matured DCs (77.7 %) in BDIR NPs + L treatment, which was 2.0-fold of that for PDT alone (Figs. S7A and B). These data verified that BDIR NPs could effectively enhance tumor immunogenicity, thereby activating stronger immune responses.

### 3.5 Tumor accumulation of BDIR NPs *in vivo*

**Fig. 3:**
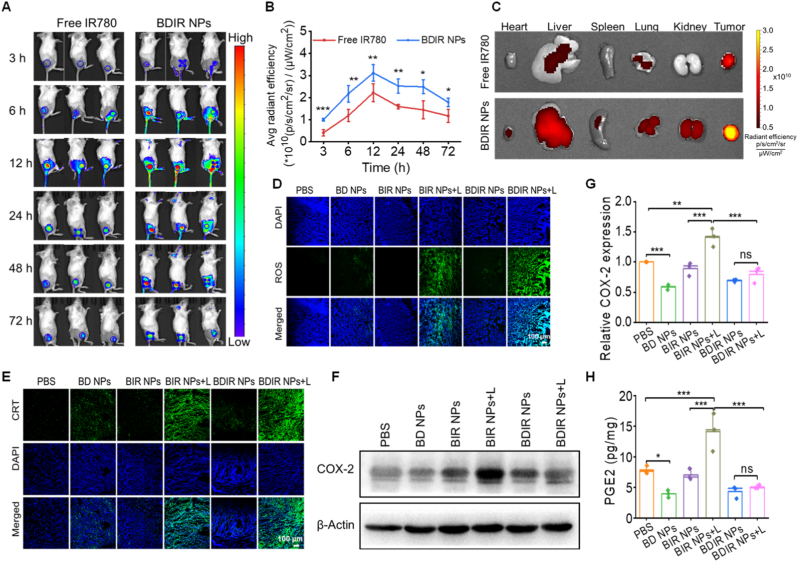
***In vivo* studies of BDIR NPs.** (A) Time-resolved IVIS imaging of tumor-bearing mice treated with free IR780 and BDIR NPs at a dose of 2 mg/kg IR780. (B) Quantification of IR780 signal at tumor site *via* IVIS imaging (n = 3). (C) Representative fluorescence imaging of the tumors and main organs at 72 h. (D) CLSM images of intratumoral ROS generation. Scale bar = 100 μm. (E) CLSM images showing CRT exposure in tumors treated with different formulations. Scale bar = 100 μm. (F) Western blot image showing COX-2 expression and (G) the quantification of COX-2 expression in the 4T1 tumors treated with different formulations (n = 3). (H) PGE2 level measured in tumor homogenates treated with different formulations (n = 3). Results are expressed as the mean ± SD, statistical significance was determined by one-way ANOVA, ∗*p* < 0.05, ∗∗*p* < 0.01, ∗∗∗*p* < 0.001, ns: no significance.

The biodistribution of BDIR NPs *in vivo* was tracked by fluorescence imaging of IR780 in tumor-bearing mice at various time points. BDIR NPs exhibited significantly stronger fluorescence compared to free IR780 from 3 to 72 h post injection (Fig. 3A and B). This could be attributed to the tumor-targeting ability of NPs facilitated by the EPR effect and potential active targeting of albumin in BDIR NPs, such as the binding with highly expressed proteins in tumors, including gp60 and secreted protein acidic and rich in cysteine (SPARC) [50]. *Ex vivo* imaging at 72 h post injection revealed fluorescence signals predominantly in the tumor (Fig. 3C), with BDIR NPs showing 2.2-fold intensity compared to free IR780 (Fig. S8). These findings demonstrated the favorable biodistribution and tumor-targeting capability of BDIR NPs.

### 3.6 ROS generation, ICD induction and COX-2/PGE2 inhibition *in vivo*

Followed by tumor accumulation, BIR and BDIR NPs effectively generate abundant ROS at the tumor site under NIR irradiation (Fig. 3D & S9), showcasing the PDT potential *in vivo*. Similar to the *in vitro* study, DCF further enhanced the ROS generation by PDT. Such ROS production elicited effective ICD *in vivo*. As expected, BIR NPs + L and BDIR NPs + L treatments were able to trigger apparently more CRT exposure compared with the counterparts without NIR irradiation (Fig. 3E & S10). In addition, thanks to DCF, BDIR NPs considerably increased the CRT exposure compared to BIR NPs, likely attributable to the enhanced ROS production facilitated by DCF, as demonstrated *in vitro* and *in vivo*. These data suggested the best capability of BDIR NPs + L treatment in activating the anti-tumor immune responses *via* ICD induction.

The COX-2/PGE2 pathway regulation was also evaluated *in vivo*. BD NPs notably inhibited COX-2 expression, wherein PDT alone (BIR NPs + L) led to a COX-2 expression elevated by 46.9 % in comparison with PBS (Fig. 3F and G), correlating with high concentrations of PGE2 in tumors (Fig. 3H). More importantly, PDT-boosted COX-2 expression was reversed by DCF in BDIR NPs, while the similarly improved PGE2 secretion was efficiently downregulated by 63.4 %, even lower than that of PBS control. These data indicated an intriguing potential for BDIR NPs to mitigate the PDT-bolstered immunosuppression induced by correlated COX-2 and PGE2 production *in vivo*, which would be desirable for PDT-provoked antitumor immunity.

### 3.7 Anti-tumor effect in bilateral tumor model

**Fig. 4:**
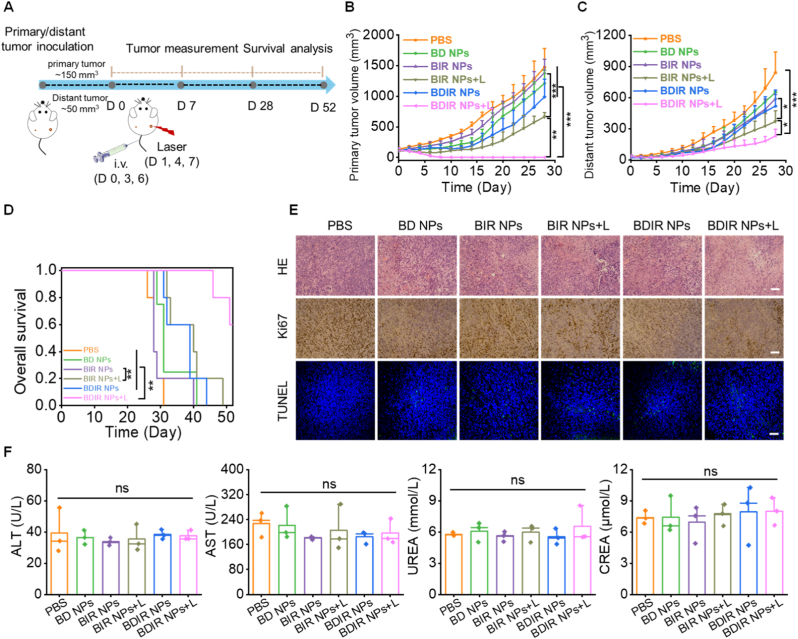
***In vivo* anti-tumor effect in bilateral 4T1 tumor model.** (A) Schematic diagram of the treatment schedule for bilateral 4T1 tumor model (IR780, 2 mg/kg; DCF, 20 mg/kg; +L: 808 nm laser, 1W/cm^2^). Growth profiles of (B) primary tumor, (C) distant tumor and (D) survival rate change of mice treated with different formulations (n = 5). Statistical significance was determined by log-rank test, ∗∗*p* < 0.01. (E) H&E, Ki-67 and TUNEL staining of 4T1 tumors treated with different formulations. Scale bar: 100 μm. (F) Blood biochemistry of ALT, AST, UREA and CREA of mice at the end of anti-tumor experiment (n = 3). Results are expressed as the mean ± SD, statistical significance was determined by one-way ANOVA, ∗*p* < 0.05, ∗∗*p* < 0.01, ∗∗∗*p* < 0.001, ns: no significance.

To assess the anti-tumor and abscopal effects of various formulations, the bilateral subcutaneous 4T1 breast cancer model was established (Fig. 4A). In the absence of NIR irradiation, tumors treated with BIR NPs and BDIR NPs revealed rapid growth comparable to that of PBS control (Fig. 4B and C). Notably, three rounds of BDIR NPs injection and laser irradiation showed sensational primary tumor eradiation for all mice and the best inhibition of distant tumors, and significantly extended the animal survival duration (Fig. 4D), suggesting a remarkable systemic therapeutic performance. For further mechanism investigation, H&E staining of tumor sections revealed that BDIR NPs + L treatment induced the most severe necrosis and nuclear ablation of tumor cells compared to other groups (Fig. 4E). Concurrently, Ki-67 staining showed the minimal proportion of proliferative tumor cells, as shown by the least brown cells, while TUNEL staining displayed the most proportion of apoptotic levels after the same treatment (Fig. 4E). Collectively, these data evidenced the capability of BDIR NPs to eradicate aggressive tumor growth through prohibiting cell proliferation and accelerating the apoptosis. Furthermore, body weight monitoring of mice treated with various formulations revealed a slow growth trend without obvious variation among diverse groups (Fig. S11). These formulations also caused no noticeable damage to major organs including heart, liver, spleen, lung, and kidney in the H&E staining (Fig. S12). In addition, the blood biochemical tests of tumor-bearing mice revealed no significant variation (*p* > 0.05) among all groups in the levels of aspartate aminotransferase (AST), alanine transaminase (ALT), blood urea and creatinine (CREA) (Fig. 4F). These data jointly suggested a promising biosafety of BDIR NPs without causing noticeable adverse effects.

### 3.8 DC maturation and re-modulation of immunosuppression *in vivo*

**Fig. 5:**
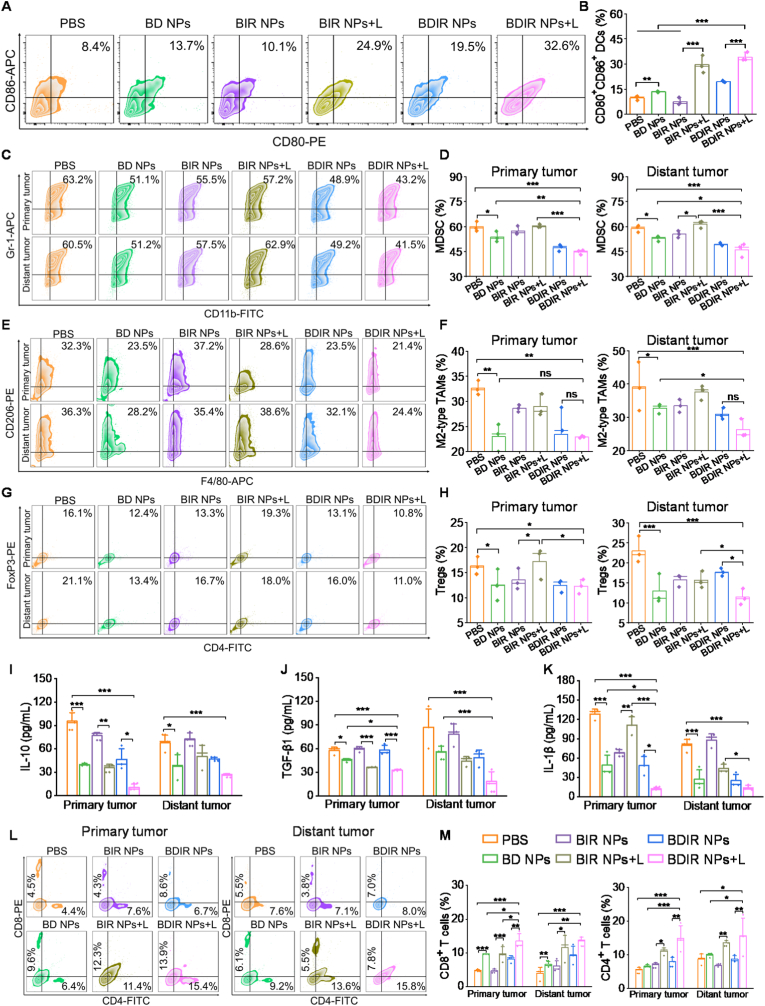
**Immune responses aroused by BDIR NPs in bilateral 4T1 tumor model**. (A) Representative flow cytometry plots of mature DCs, and (B) quantification of mature DCs marked as CD80^+^CD86^+^ cells in TDLNs. (C) Representative flow cytometry plots of MDSCs, and (D) the infiltration quantification in primary and distant tumors. (E) Representative flow cytometry plot of M2-type TAMs, and (F) the infiltration quantification in primary and distant tumors. (G) Representative flow cytometry plots of Tregs, and (H) the infiltration quantification in primary and distant tumors. The quantification of (I) IL-10, (J) TGF-β1, and (K) IL-1β levels in primary and distant tumors following the indicated treatments. (L) Representative flow cytometry plots of CD4⁺ and CD8⁺ T cells and (M) their infiltration quantifications in primary and distant tumors receiving various treatments. Results are expressed as the mean ± SD. (n = 3, statistical significance was determined by one-way ANOVA, ∗*p* < 0.05, ∗∗*p* < 0.01, ∗∗∗*p* < 0.001, ns: no significance).

To investigate the immune mechanisms underlying the dramatic anti-tumor effect of BDIR NPs, the tumor-draining lymph nodes (TDLN) and tumors were collected after 7 days post-treatment and analyzed through flow cytometry. DC maturation was firstly detected in TDLNs (Fig. 5A and B, S13A), whereas the fractions of matured DCs (CD80^+^CD86^+^) in BD NPs, BIR NPs + L, BDIR NPs and BDIR NPs + L-treated mice 1.4-, 3.0-, 2.1- and 3.6-fold of that for the PBS group, indicating BDIR NPs + L maximally promoted DC maturation, which could trigger the developments of CTLs for anti-tumor immunity. Next, we evaluated the re-modulation of immunosuppressive cells including the tumor-infiltrating Tregs, M2-type TAMs and MDSCs. Notably, the BDIR NPs + L group showed nearly 25 % and 22 % reductions in MDSC infiltration in primary and distant tumors, respectively, compared to the PBS-treated group (Fig. 5C and D, S13B). Further verification of TAMs in tumors showed that the infiltration of M2-type TAMs (CD11b^+^F4/80^+^CD206^+^) was efficiently decreased in both tumors following BDIR NPs + L treatment (Fig. 5E and F, S13C). Additionally, the proportion of Tregs (CD4^+^FoxP3^+^) in tumors receiving the same treatment exhibited a significant decrease compared to PBS and BIR NPs + L groups (Fig. 5G and H, S13D). The significantly attenuated infiltration of these major immunosuppressive cells could be attributed to efficient COX-2/PGE2 pathway suppression by DCF demonstrated above (Fig. 3F–H).

MDSCs can activate their immunosuppressive effects by secreting abundant quantities of immunosuppressant cytokines like IL-10 and TGF-β1 [51]. Additionally, TAMs produce IL-10 and IL-1β, which can enhance COX-2 expression in breast cancer cells, thereby promoting the advancement of cancer [52]. Consequently, the intratumoral secretion of IL-10, IL-1β, and TGF-β1 was examined by ELISA. It's found that these immunosuppressive cytokines in both primary and distant tumors could be further suppressed in BDIR NPs + L-treated tumors (Fig. 5I–K). Finally, the downregulated immunosuppressive cells and cytokines, as well as increased mature DC, contributed to a more immune-supportive TME, as demonstrated by apparently enhanced presence of CD8^+^ and CD4^+^ T cells in both tumors of BDIR NPs+L group (Fig. 5L and M, S13E). CTLs could secrete IFN-γ and TNF-α, increase antigen presentation and support CTL efficacy, thus bolstering anti-tumor immunity [53]. In agreement with this, an obviously upregulated expression of TNF-α and IFN-γ was observed by BDIR NPs + L treatment (Figs. S14A and B). These data forcefully indicated the improvement of systemic anti-tumor immunity triggered by the ICD induction by PDT and minorly by DCF, and the efficient immunosuppression remodulation by DCF. More importantly, these data shed reasonable light on the boosted bilateral tumor treatment outcomes and abscopal effects of BDIR NPs assisted by NIR irradiation (Fig. 4B–D).

### 3.9 Suppression of tumor relapse and metastasis

**Fig. 6:**
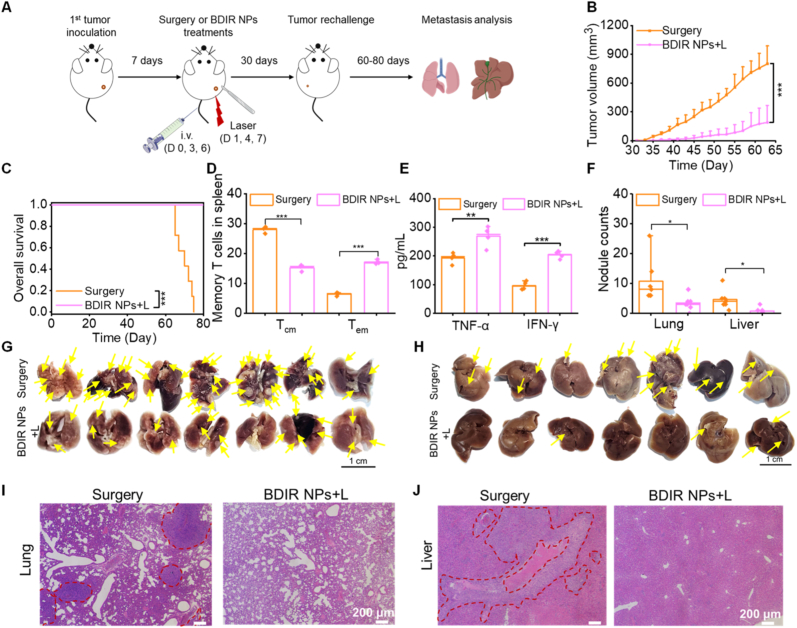
**Immune memory effect provoked by BDIR NPs + L treatment.** (A) Schematic diagram of the treatment schedule in rechallenged tumor model. (B) Rechallenged tumor volume changes in mice receiving the surgery or BDIR NPs + L treatments for the first tumor (n = 7). (C) Overall survival profile of mice bearing rechallenged tumors (n = 7). Statistical significance was determined by log-rank test, ∗∗∗*p* < 0.001. (D) Frequency of effector memory T cells (T_em_) and central memory T cells (T_cm_) in spleens of mice treated by surgery or BDIR NPs + L (n = 3). (E) TNF-α and IFN-γ levels in sera of mice 7 days post tumor rechallenge (n = 4). (F) Number of metastatic tumor nodules in lung and liver of rechallenged mice following surgery and BDIR NPs + L treatments (n = 7) and the photographs of lung (G) and (H) liver metastasis. Scale bar = 1 cm. The yellow arrows partially highlight the locations of metastasis for better visualization. H&E staining of lungs (I) and livers (J) sections showing the metastatic tumor nodules in rechallenged mice following surgery and BDIR NPs + L treatments. Scale bar = 200 μm. Metastasis are indicated by dashed circles. Results are expressed as the mean ± SD, statistical significance was determined by one-way ANOVA, ∗*p* < 0.05, ∗∗*p* < 0.01, ∗∗∗*p* < 0.001.

Afterwards, the rechallenged tumor model was established to assess long-term protection against tumor recurrence and metastasis (Fig. 6A). BDIR NPs + L treatment greatly inhibited rechallenged tumor growth compared to the surgery treatment (Fig. 6B). Even though 4 of 7 mice treated with BDIR NPs + L revealed tumor recurrence after 10 days of rechallenge (Fig. S15), these rechallenged tumors were significantly smaller than the surgery group, which resulted in much extended animal survival (Fig. 6C). To investigate the mechanism behind this, the spleens of mice were analyzed for memory T cells *via* flow cytometry before the tumor rechallenge. Intriguingly, compared to surgery-treated mice, an obvious decrease of central memory T cells (T_cm_: CD3^+^CD8^+^CD44^+^CD62L^+^) in CD8^+^ T cells and an increase of effector memory T cells phenotype (T_em_: CD3^+^CD8^+^CD44^+^CD62L^−^) were observed in long-term surviving mice upon BDIR NPs + L treatment (Fig. 6D). This shift from T_cm_ to T_em_ phenotype was comparable to the literature reports [32,54]. The obviously augmented suppression of tumor relapse and extended animal survival could be ascribed to such transition, especially considering the critical role of memory T cells including T_em_ in long-term tumor regression [9,55]. Besides, T_em_ could directly trigger robust immunological defense through secreting anti-tumor cytokines like TNF-α and IFN-γ [56,57]. Therefore, their concentrations in the serum of mice were quantified by ELISA seven days post the rechallenge tumor inoculation. Indeed, the serum TNF-α and IFN-γ levels significantly increased by ∼40 % and 100 %, respectively in mice treated with BDIR NPs + L compared to surgery resection (Fig. 6E). Jointly, these findings provided crucial evidences for the establishment of strong anti-tumor immune memory by BDIR NPs + L treatment. Moreover, tumor metastasis in the lungs and livers of BDIR NPs + L-treated mice was also significantly suppressed even at 40 days after the tumor rechallenge, as shown by much less metastatic nodules (3.4 ± 2.4 in lungs; 0.7 ± 1.1 in livers) compared to surgery group (10.7 ± 7.3 in lungs; 4.6 ± 3.2 in livers) with metastatic outgrowth (Fig. 6F–H). Furthermore, H&E staining of lung and liver sections revealed numerous metastatic nodules in the surgery groups, while minimal or no metastatic regions were observed in those treated with BDIR NPs + L (Fig. 6I and J). Taken together, these data implied that BDIR NPs + L activated robust immune memory effects, thereby preventing tumor recurrence and metastasis in the lungs and livers.

### 3.10 Anti-tumor efficacy of PD-L1 blockade boosted by BDIR NPs in bilateral 4T1 tumor model

**Fig. 7:**
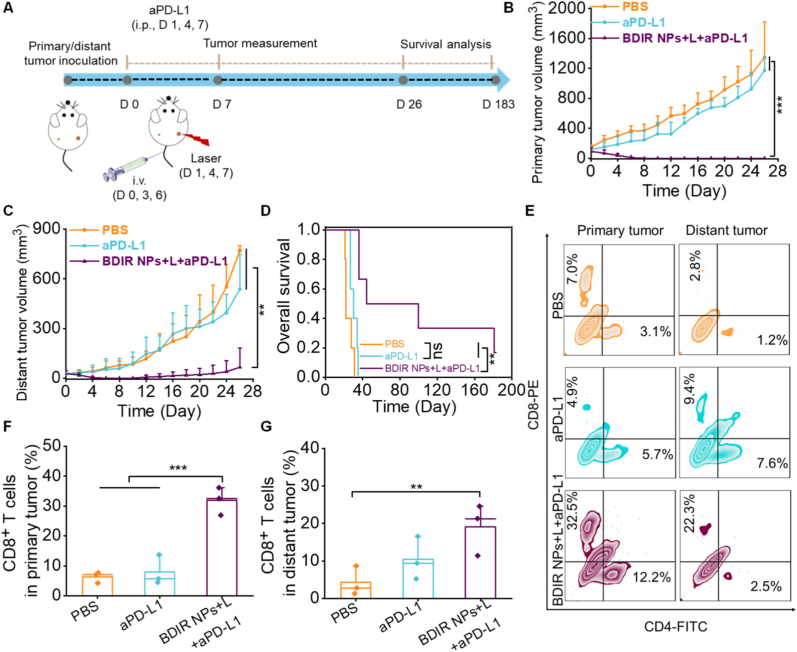
***In vivo* anti-tumor effects of BDIR NPs combined with aPD-L1 treatment.** (A) Schematic illustration of BDIR NPs and aPD-L1 treatment. (B, C) Primary and distant tumor volume profile, and (D) survival curve of BALB/c mice treated with different formulations (n = 5). Statistical significance was determined by log-rank test, ∗∗*p* < 0.01, ns: no significance. (E) Representative flow cytometry plots showing the percentage of CD4^+^ and CD8^+^ T cells in primary and distant tumors. CD8^+^ T cells level in (F) primary and (G) distant tumors (n = 3). Results are expressed as the mean ± SD, statistical significance was determined by one-way ANOVA, ∗*p* < 0.05, ∗∗*p* < 0.01, ∗∗∗*p* < 0.001.

The 4T1 tumors are commonly considered poorly immunogenic and less responsive to ICB therapy [13]. Given this, BDIR NPs were examined in bilateral 4T1 tumor-bearing mice to verify whether they could energize PD-L1 blockade (aPD-L1), while the mice treated with PBS and aPD-L1 were used as control (Fig. 7A). Compared to the PBS group, PD-L1 blockade resulted in a modest delay in tumor growth in both primary and distant tumors, with no further benefit for mice survival, which was 35 days for aPD-L1 treatment and 31 days for PBS treatment (Fig. 7B–D). However, the combination of BDIR NPs and aPD-L1 greatly impaired the tumor progression, with complete inhibition of primary tumor growth and pronounced delay in distant tumor growth. This combination treatment also apparently extended the survival time. Notably, almost 50 % of mice treated with this combination survived for at least 100 days, whereas 80 % of mice exempted from any primary or distant tumors (Fig. S16C). In contrast, there was a lack of distant tumor growth control in the mice treated with PBS and PD-L1 antibody alone (Figs. S16A and B). The effect of the above 3 different treatments on the tumor-infiltrating immune cells in 4T1 tumor-bearing mice was analyzed. Impressively, the intratumoral CD8^+^ T cell infiltration in primary tumor was increased by 2.3 or 4.6 folds, respectively in BDIR NPs + aPD-L1-treated mice under NIR irradiation compared with aPD-L1 or PBS-treated mice (Fig. 7E and F), while that of CD4^+^ T cells increased by 4.8 or 8.1 folds (Fig. S17A), respectively. The combination therapy also contributed to an obvious increase of CD8^+^ and CD4^+^ T cells in distant tumors (Fig. 7G & S17B). These results implied that BDIR NPs could effectively augment the effectiveness of PD-L1 blockade therapy for TNBCs *via* boosting the effector T cell infiltration.

## 4 Conclusions

In summary, we have successfully developed a biocompatible albumin nanoplatform to combine PDT and COX-2/PGE2 inhibition for enhanced photodynamic immunotherapy. The resultant NPs displayed a favorable average size, stability, remarkable ROS production capability, and desirable drug release for immune re-modulation. They could effectively enter 4T1 cells, generate ROS upon NIR irradiation, and stimulate powerful immune responses by inducing tumor ICD. Meanwhile, diclofenac-related inhibition of COX-2/PGE2 pathway could reshape the tumor immunosuppression deteriorated by PDT majorly *via* downregulating the intratumoral immunosuppressive cells including MDSCs, Tregs and M2-type TAMs, and decreasing the production of immunosuppressive cytokines such as IL-10, IL-1β, and TGF-β1 to reinforce the antitumor immunity. Consequently, such synergistic therapy achieved an elimination of primary tumors, effective suppression of distant tumors *via* abscopal effect, and a resultful prevention of tumor recurrence and metastasis in the lungs and livers of mice. Furthermore, the combination regimen of BDIR NPs and anti-PD-L1 antibody demonstrated distinguished synergy in suppressing tumor progression owing to enhanced intratumoral infiltration of CTLs. This strategy overcomes the immunosuppressive drawbacks of PDT, and sets an intriguing example for improving the resultant cancer immunotherapy. Moreover, given the upregulated COX-2 expression widely caused by chemotherapy and other ROS-producing therapies (e.g., radiotherapy) [13,27], it also offers constructive insights to enhance the synergistic immunotherapy potentiated by these clinical treatments.

## CRediT authorship contribution statement

**Tao Xu:** Writing – original draft, Methodology, Investigation, Formal analysis, Data curation, Conceptualization. **Kehan Liu:** Investigation, Data curation. **Shuqi Mi:** Investigation, Data curation. **Yao Yao:** Investigation. **Mengyao Zhang:** Investigation. **Shujuan Xue:** Investigation. **Feng Zhi:** Writing – review & editing, Supervision, Funding acquisition, Conceptualization. **Sally-Ann Cryan:** Writing – review & editing, Supervision, Funding acquisition, Data curation, Conceptualization. **Dawei Ding:** Writing – review & editing, Supervision, Funding acquisition, Data curation, Conceptualization.

## Declaration of competing interest

The authors declare no conflicts of interest.

## Data Availability

Data will be made available on request.
