# Supplementary material for: Cyclooxygenase-2/prostaglandin E2 inhibition remodulated photodynamic therapy-associated immunosuppression for enhanced cancer immunotherapy
Source: Mater Today Bio. 2025 Jan 28;31:101530. doi: 10.1016/j.mtbio.2025.101530 (PMC11847551; doi:10.1016/j.mtbio.2025.101530)
Supplement: Multimedia component 1 [file mmc1.pdf]

Supplementary Information

Cyclooxygenase-2/Prostaglandin E2 Inhibition Remodulated Photodynamic Therapy-Associated Immunosuppression for Enhanced Cancer Immunotherapy

Tao Xu[1,2], *Kehan Liu*[1], *Shuqi Mi*[1], *Yao Yao*[3], *Mengyao Zhang*[1], *Shujuan Xue*[1], *Feng Zhi*[4,5,*], *Sally-Ann Cryan* [2,*], *Dawei Ding*[1,6,7*]

[1]College of Pharmaceutical Sciences, Soochow University, Suzhou 215123, China

[2]School of Pharmacy and Biomolecular Sciences, Royal College of Surgeons in Ireland (RCSI), Dublin D02 YN77, Ireland

[3]Department of Gerontology, The Affiliated Suqian Hospital of Xuzhou Medical University, Suqian 223800, China

[4]Department of Neurosurgery, The First People's Hospital of Changzhou, Changzhou 213003, China

[5]Clinical Medical Research Center, The Third Affiliated Hospital of Soochow University, Changzhou 213003, China

[6]Wisdom Lake Academy of Pharmacy, Xi'an Jiaotong-Liverpool University, Suzhou 215123, China

[7]Jiangsu Province Higher Education Key Laboratory of Cell Therapy Nanoformulation (Construction), Xi'an Jiaotong-Liverpool University, Suzhou 215123, China

*The correspondence should be addressed to F. Zhi (danielzhif@suda.edu.cn), S. Cryan (SCryan@rcsi.ie) and D. Ding (Dawei.Ding@xjtlu.edu.cn).

Experimental Section

Materials

Bovine serum albumin (BSA, ≥98%), IR780 iodide (IR780) and Millipore ultra-centrifugal filters (100 kD) were ordered from Sigma-Aldrich (St. Louis, Missouri, USA). Diclofenac (DCF, ≥99%) was obtained from Meilune Biotechnology (Dalian, China). Singlet oxygen sensor green (SOSG) was purchased from Thermo Fisher Scientific (Dublin, Ireland). 2'-7'dichlorofluorescin diacetate (DCFH-DA), 4',6-diamidino-2-phenylindole (DAPI), ATP assay kit, FITC-labeled goat anti-rabbit IgG and Alexa Fluor 488-labeled goat anti-rabbit IgG were purchased from Beyotime (Shanghai, China). Cell Counting Kit-8 (CCK-8), protease inhibitor cocktails (K1024) and phosphatase inhibitor cocktails (K1015) were obtained from APExBIO (Shanghai, China). Calreticulin (CRT) rabbit monoclonal antibody (mAb) (27298-1-AP) was purchased from Proteintech (Wuhan, China). COX-2/PTGS2 rabbit mAb (A3560), β-Actin Rabbit mAb (AC038), HRP-conjugated goat anti-rabbit IgG heavy chain (AS063) were purchased from ABclonal (Wuhan, China). RIPA Lysis Buffer (PC101) was obtained from Epizyme (Shanghai, China). BCA assay kit, enhanced chemiluminescence kit (ECL kit), one-step PAGE gel fast preparation kit (10%) and Annexin V-FITC/PI apoptosis detection kit was obtained from Vazyme (Nanjing, China). Flow cytometry antibodies including FITC anti-mouse CD11c, PE anti-mouse CD80, APC anti-mouse CD86, Percp/Cy5.5 anti-mouse CD3, PE anti-mouse CD8a, FITC anti-mouse CD4,Percp/Cy5.5 anti-mouse CD3, FITC anti-mouse CD4, APC anti-mouse CD25, PE anti-mouse FOXP3, PE anti-mouse CD45, FITC anti-mouse CD11b, APC anti-mouse Gr1, FITC anti-mouse CD11b, APC anti-mouse F4/80, PE/Cyanine7 anti-mouse CD86 Antibody, PE anti-mouse CD206, FITC anti-mouse CD3, PE anti-mouse/human CD44, Percp/Cy5.5 anti-mouse CD8a, APC anti-mouse CD62L and True-Nuclear™ Transcription Factor Buffer Set were purchased from Biolegend (San Diego, CA, USA). Collagenase IV and cell strainers 70 μm cell strainers were purchased from Biosharp (Beijing, China), DNAase was obtained from Aladdin (Shanghai, China), Hyaluronidase (HAase) was purchased from Sigma (Shanghai, China). ELISA kits of mouse PGE2, HMGB1, IL-10, TGF-β1, IL-1β, TNF-α and IFN-γ were obtained from Animaluni (Shanghai, China). Anti-mouse PD-L1 monoclonal antibody (B7-H1) (10 mg/mL) was

purchased from BioXCell (West Lebanon, NH, USA). 4T1 cells and DC2.4 cells were cultured at 37°C and 5% CO_2 in RPMI 1640 medium (Procell, Wuhan, China), supplemented with 10% fetal bovine serum (Sigma, St. Louis, Missouri, USA) and 1% penicillin/streptomycin (Beyotime, Shanghai, China).

Characterizations of BDIR NPs

The content of IR780 and diclofenac in BDIR BPs were determined by two methods. The absorbance of IR780 was measured at 780 nm using UV-vis spectroscopy (Shimadzu UV-2600, Japan), while diclofenac was quantified at 276 nm through high-performance liquid chromatography (HPLC, Agilent 1260, USA). The concentration of BSA was determined by BCA assay kit for the quantification of drug loading content (DL%) and drug encapsulation efficiency (EE%) of both drugs. The size and morphology were analyzed using dynamic light scattering (DLS, Zetasizer Nano ZS90, England) and transmission electron microscopy (TEM, Hitachi HT7700, Japan). Briefly, the drug-loaded BSA NPs dispersion was diluted with deionized water, and then DLS was used to determine the hydrodynamic diameter (D_h), polydispersity index (PDI), and zeta potential (ZP), while TEM was used to observe the morphology and size in dehydrated state. For the colloidal stability study, the BDIR NPs were dispersed into PBS buffer (pH 7.4) and incubated at 4°C for 7 days, while D_h was measured every day using DLS. The Fourier-transform infrared (FTIR) spectra of BSA, IR780, DCF and BDIR NPs were recorded with a Bruker Vertex 70+ Hyperion 2000 instrument (USA), acquiring data in the range of 600–4000 cm^{-1} with a resolution of 2 cm^{-1}. The samples were analyzed in solid forms of raw powder or lyophilized NPs. Photostability was also evaluated. Briefly, free IR780, BIR NPs and BDIR NPs (5 μg/mL IR780, 2 mL for each) in PBS were irradiated by 808 nm light exposure at 0.5 W/cm^2 for 5 minutes, while the absorptions were measured with a UV-vis spectrophotometer every 30 s.

The *in vitro* release of DCF and IR780 from BDIR NPs at 37°C was carried out using a dialysis method under different pH conditions from 7.4 (pH of blood) to 6.8 (that of TME) and 5.5 (lysosomal environment). BDIR NPs solution was aliquoted in a number of dialysis bags (COMW: 3500 Da), which were then incubated in 50 ml tubes with 20 mL buffers containing 0.5% (v : v) tween 80, and placed in a shaker incubator (100 rpm) at 37°C (*n*=3).

At determined time points, 1 mL of the release buffer was removed and replenished with an equal volume of fresh medium. The concentration of the released DCF and IR780 were analyzed using HPLC and UV-Vis as above.

Cellular uptake and intracellular distribution in 4T1 cells

Intracellular uptake was firstly evaluated through observing the fluorescence accumulation of IR780. Briefly, 4T1 cells were seed into 24-well glass bottom plate at a density of 2×10^4 cells per well and cultured overnight. Afterwards, the cells were treated with BDIR NPs and free IR780 (1 μg/mL IR780) for 6 h, 12 h and 24 h. After treatment, they were washed with PBS and fixed in 4% PFA for 15 min. After washing, the cells were stained with DAPI (10 μg/mL, 200 μL) for 10 min at 37°C in the dark, followed by imaging with a confocal laser scanning microscope (CLSM, Nikon A1R HD25, Japan) for fluorescence imaging. Cellular uptake of IR780 was also quantified by flow cytometry. 4T1 cells (2×10^5 cells/well) were plated in 6-well plates with 2 mL of media, and treatments were conducted as mentioned above. Following the treatments, the wells were washed with PBS twice, gently dissociated from the wells with trypsin, and then resuspended in PBS. The flow cytometry (FC, BD ARAIII, USA) was employed to quantitatively analyze the fluorescence intensity with 633 nm excitation and 780 nm emission.

Subcellular localization of BDIR NPs in 4T1 cells and the ROS damage membrane effect were detected according to a previously established protocol [1]. Briefly, commercially available LysoTracker™ Green DND-26 were used to track lysosome. After 4 h of co-culture of 4T1 cells with BDIR NPs, 50 nM LysoTracker Green DND-26 were added to label the lysosomes for 0.5 h. Following three washes with PBS, the cells were fixed and stained with DAPI (10 μg/mL, 10 min). Then, the subcellular localization was observed using CLSM. Fluorescence mages captured from the same visual field were merged for co-localization analysis.

Cellular apoptosis

Cell apoptotic level was assessed using apoptosis detection kit with Annexin V-FITC and PI. In general, 4T1 cells (1×10^5 cells per well) were plated in a 6-well plate for attachment. Following 12 h incubation, cells were treated with different formulations and NIR

irradiation as described previously. The collected cells were stained following the kit manufacturer's instruction, and analyzed using flow cytometry (FC).

Intratumoral ROS generation and COX-2/PGE2 inhibition *in vivo*

The intratumoral ROS production was evaluated by CLSM. For mice bearing single 4T1 tumors, different formulations as above were administered 2 mg/kg IR780 when the tumor size reached about 150 mm^3. After 24 h, 50 μL DCFH-DA (100 μM) were injected into the tumors. After 0.5 h, the tumors were subjected to NIR irradiation (5 min, 1.0 W/cm^2) for "+L" groups (same as below for *in vivo* studies). The tumors were extracted and frozen to prepare 8.0 μm tumor sections 6 h post-irradiation. The tumor sections were subsequently rinsed and stained with DAPI followed by the fluorescence observation. Similarly, tumor bearing mice were grouped and treated as described above, while tumors were collected, cut into small pieces and homogenized in pre-chilled homogenization buffer containing protease inhibitors. Centrifugation at a low speed (1000 *g*, 5 min) at 4°C was used to remove cellular debris. Finally, supernatant was collected for COX-2 detection *via* western blot and PGE2 determination by ELISA kit following the manufacturers' instructions.

ICD induction *in vivo*

To assess treatments-induced CRT expression *in vivo*, tumors from the different treatment groups as above were fixed in 4% PFA, embedded in optimal cutting temperature compound, and sliced to a thickness of 10 μm. These tumors slices were then incubated with CRT antibody (1:200) and FITC-labelled secondary antibody (1:500) and DAPI, respectively before the observation by CLSM.

Ex vivo histological staining, Ki-67 staining and TUNEL assay

Tumor-bearing mice were assigned to 6 groups, and treated with various formulations/irradiation for 3 rounds as above. Key organs including heart, liver, spleen, lung, kidney and tumor were collected after 7 days of three treatments. All samples were fixed, embedded with paraffin, and sliced into 5 μm thickness sections. All collected samples were stained with hematoxylin and eosin (H&E) for morphological analysis. Tumor sections were incubated with primary antibodies of anti-Ki-67 for

immunohistochemistry and anti-TUNEL for immunofluorescence examination.

Biosafety assay

Biosafety of BDIR NPs was evaluated by analyzing plasma biochemical indexes, including the sera levels of alanine transaminase (ALT), aspartate aminotransferase (AST), blood urea and creatinine (CREA). Plasma samples collected from mice treated with NPs were analyzed by using an automated biochemical analyzer (MEK-6410C, Japan).

Supplementary Figures

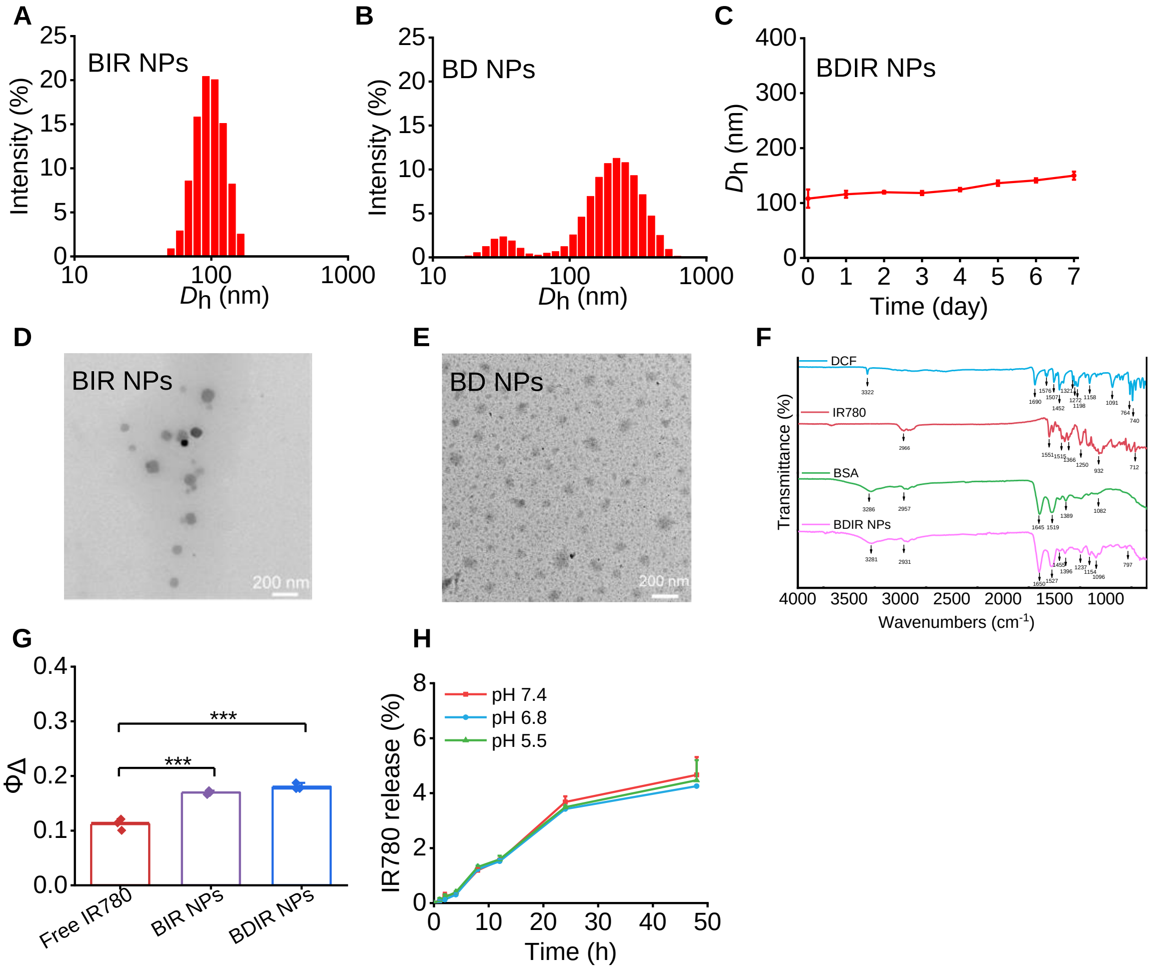

Fig. S1. Fabrication and characterization of NPs. (A) Hydrodynamic diameter (D_h) of BIR NPs and (B) BD NPs detected by DLS. (C) Size stability of BDIR NPs by DLS within one week. (D) TEM images of BIR NPs and (E) BD NPs. Scale bar: 200 nm. (F) The Fourier-transform infrared (FTIR) spectra of DCF, IR780, BSA and BDIR NPs. (G) Singlet oxygen quantum yield (Φ_Δ) of different formulations containing IR780. (H) Release profile of IR780 from BDIR NPs at different pH conditions. Results are expressed as the mean ± SD. (n=3, statistical significance was determined using Student's t-test, ***$p < 0.001$).

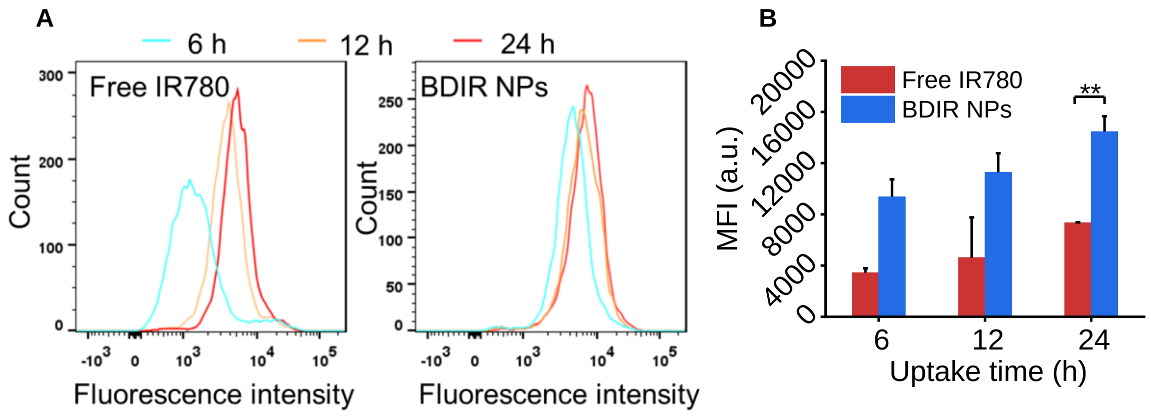

Fig. S2. Time-dependent 4T1 cellular uptake. (A) Flow cytometry chart showing the cellular uptake of free IR780 and BDIR NPs by 4T1 cells. (B) Mean fluorescence intensity (MFI)-based quantification of cellular uptake for free IR780 and BDIR NPs. Results are expressed as the mean ± SD. (n=3, statistical significance was analyzed using Student's t-test. ***p* < 0.01).

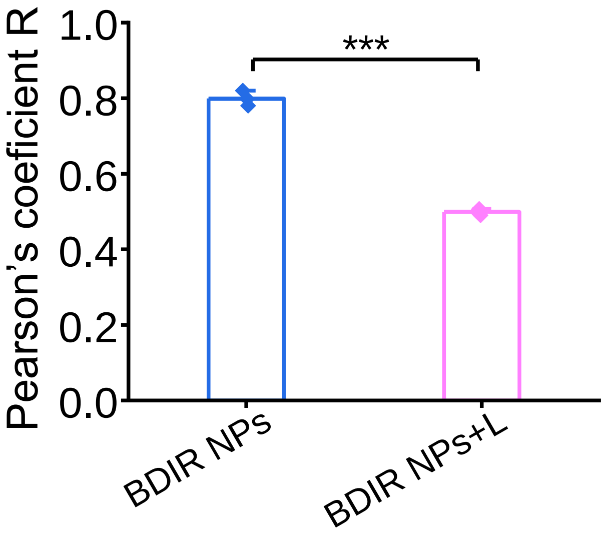

Fig. S3. Quantification of Pearson's coefficient correlation between BDIR NPs and lysotracker using ImageJ. Results are expressed as the mean ± SD. (n=3, statistical significance was analyzed using Student's t-test. ***$p < 0.001$).

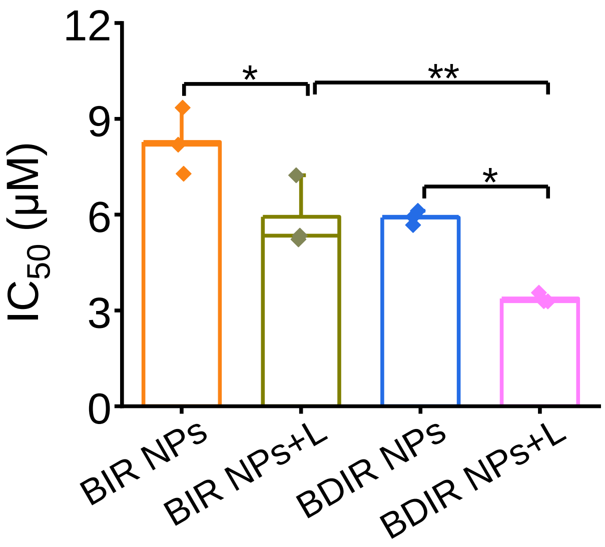

Fig. S4. Calculated IC_{50} of IR780 in various formulations against 4T1 cells. Results are expressed as the mean ± SD. (n=3, statistical significance was analyzed using one-way ANOVA. **p* < 0.05, ***p* < 0.01).

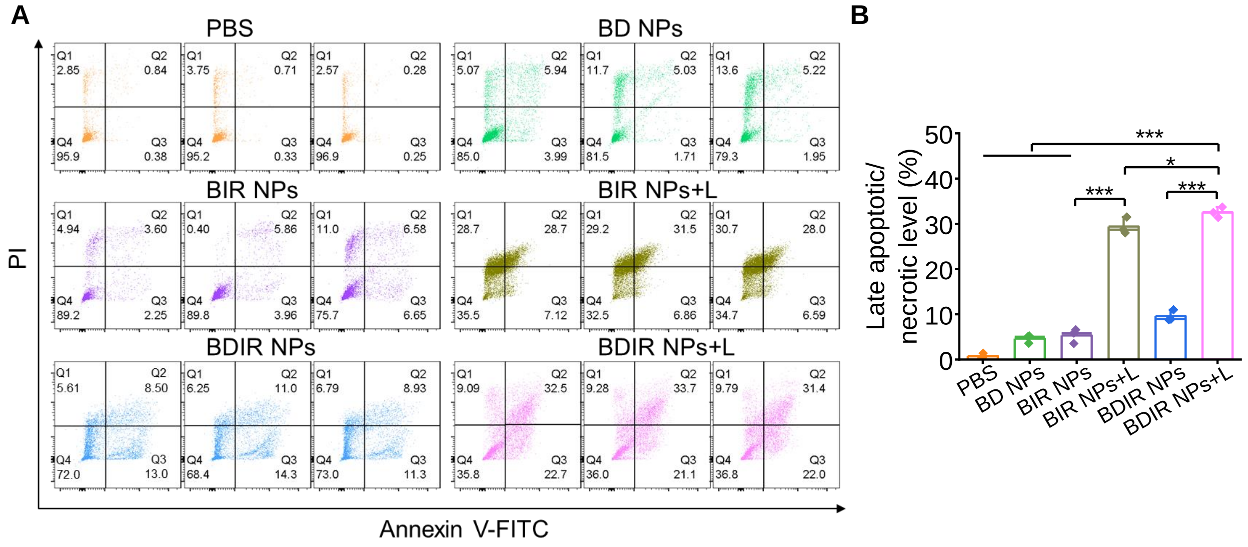

Fig. S5. Annexin V-FITC/PI staining. (A) Flow cytometry plot of Annexin V-FITC/PI staining in 4T1 cells. (B) Quantification of late-stage apoptosis/necrosis after the treatment with various formulations. Results are expressed as the mean ± SD. (n=3, statistical significance was analyzed using one-way ANOVA. *$p < 0.05$, ***$p < 0.001$).

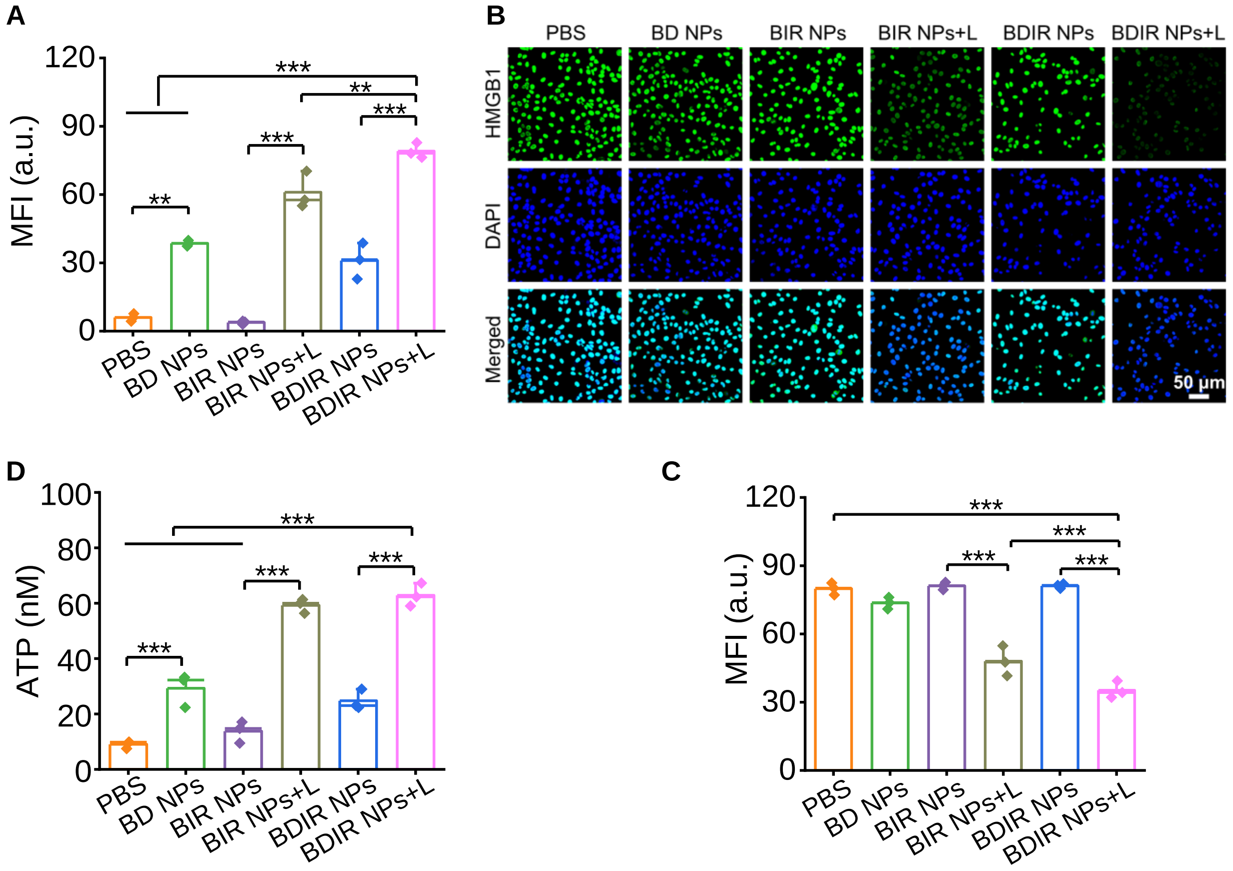

Fig. S6. *In vitro* ICD induction. (A) Semi-quantification of CRT MFI in 4T1 cells treated with different formulations by image J. (B) Confocal microscopic images of HMGB1 release. (C) Semi-quantification of HMGB1 MFI in 4T1 cells treated with different formulations by image J. (D) ATP secretion of 4T1 cells treated with different formulations. Results are expressed as the mean ± SD. (n=3, statistical significance was analyzed using one-way ANOVA. $^{**}p < 0.01$, $^{***}p < 0.001$).

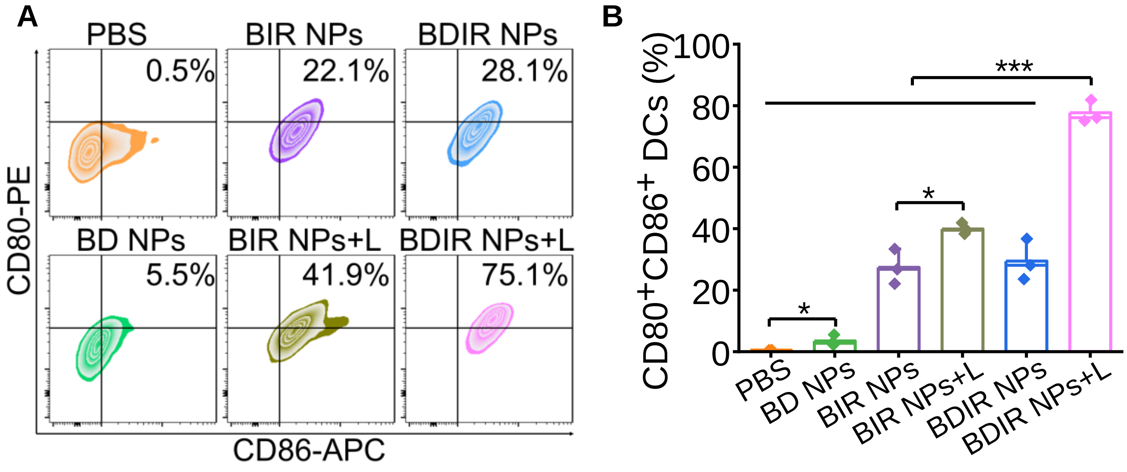

Fig. S7. DC maturation *in vitro*. (A) Flow cytometry plots showing the maturation of DC2.4 cells. (B) Quantification of mature DCs. Results are expressed as the mean ± SD. (n=3, statistical significance was determined by one-way ANOVA. *$p < 0.05$, ***$p < 0.001$).

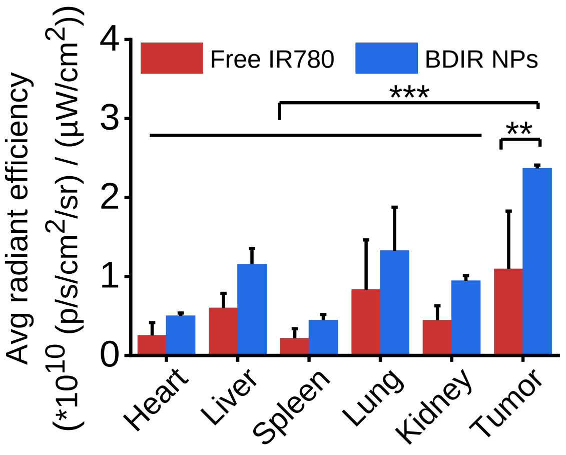

Fig. S8. Time-resolved semi-quantification of fluorescence intensity in tumors and main organs from sacrificed mice at 72 h post intravenous injection. Results are expressed as the mean ± SD. (n=3, statistical significance was analyzed using one-way ANOVA. **$p < 0.01$, ***$p < 0.001$).

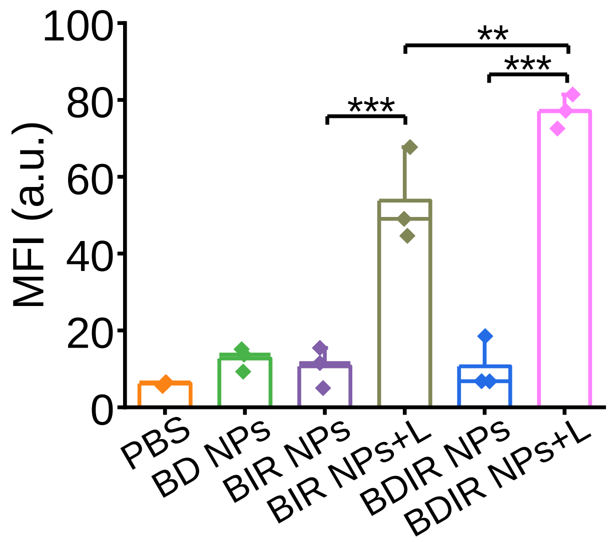

Fig. S9. Semi-quantification of mean fluorescence intensity (MFI) showing ROS generation in tumors treated with various formulations. Results are expressed as the mean ± SD. (n=3, statistical significance was analyzed using one-way ANOVA. **p < 0.01, ***p < 0.001).

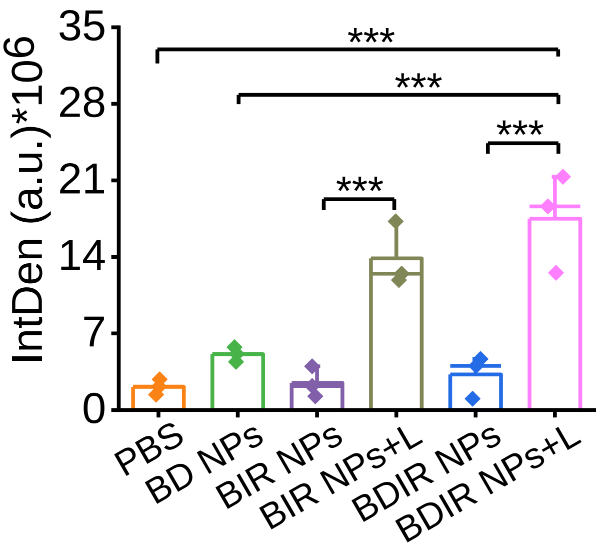

Fig. S10. Semi-quantification of fluorescence intensity showing CRT exposure in tumors treated by various formulations. Results are expressed as the mean ± SD. (n=3, statistical significance was analyzed using one-way ANOVA. ***p < 0.001)

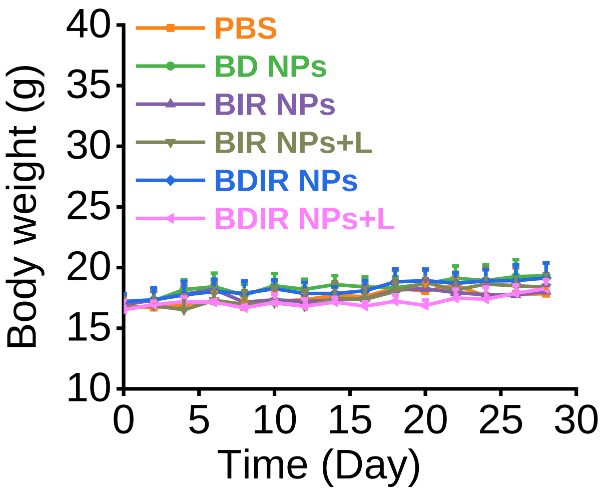

Fig. S11. Body weight change of bilateral tumor-bearing BALB/c mice treated with different formulations. Results are expressed as the mean ± SD. (n=5).

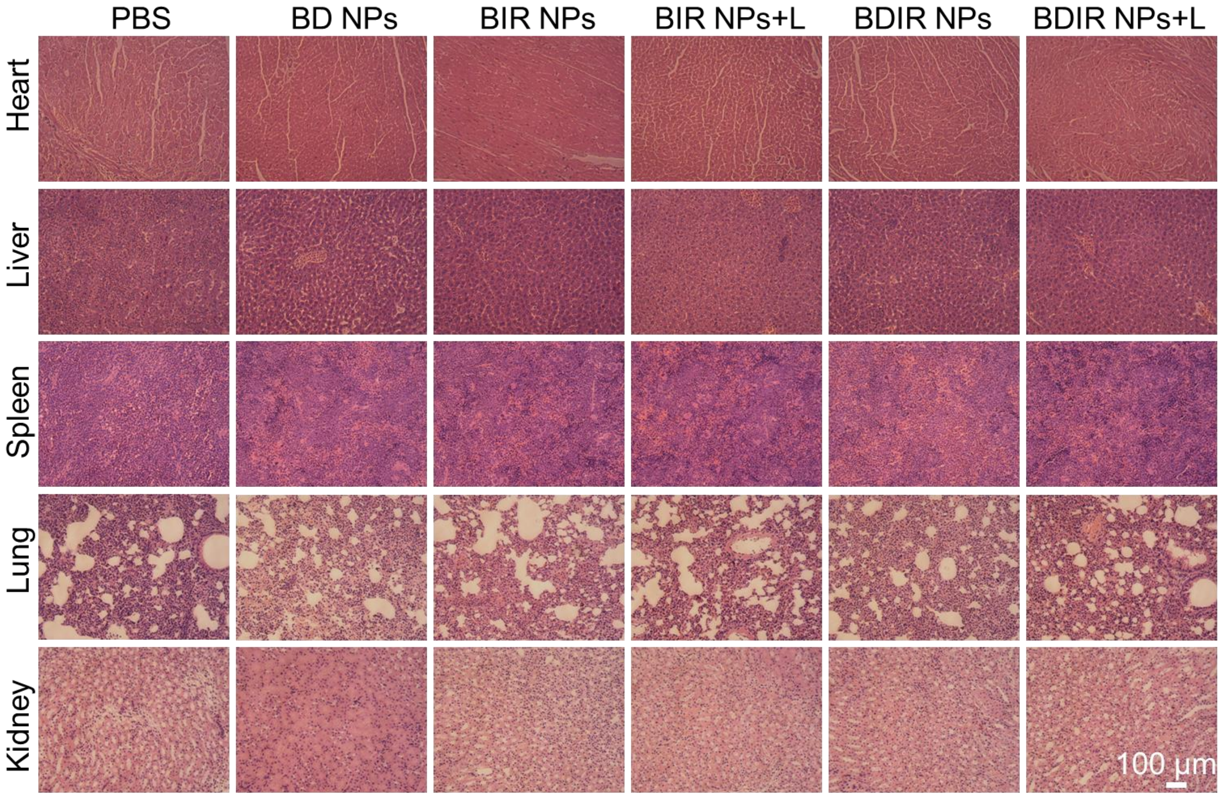

Fig. S12. H&E staining of heart, liver, spleen, lung and kidney sections of mice treated with PBS, BD NPs, BIR NPs, BIR NPs+L, BDIR NPs, and BDIR NPs+L (IR780, 2 mg/kg; DCF, 20 mg/kg; +L: 808 nm laser, 1W/cm^2). Scale bar: 100 µm.

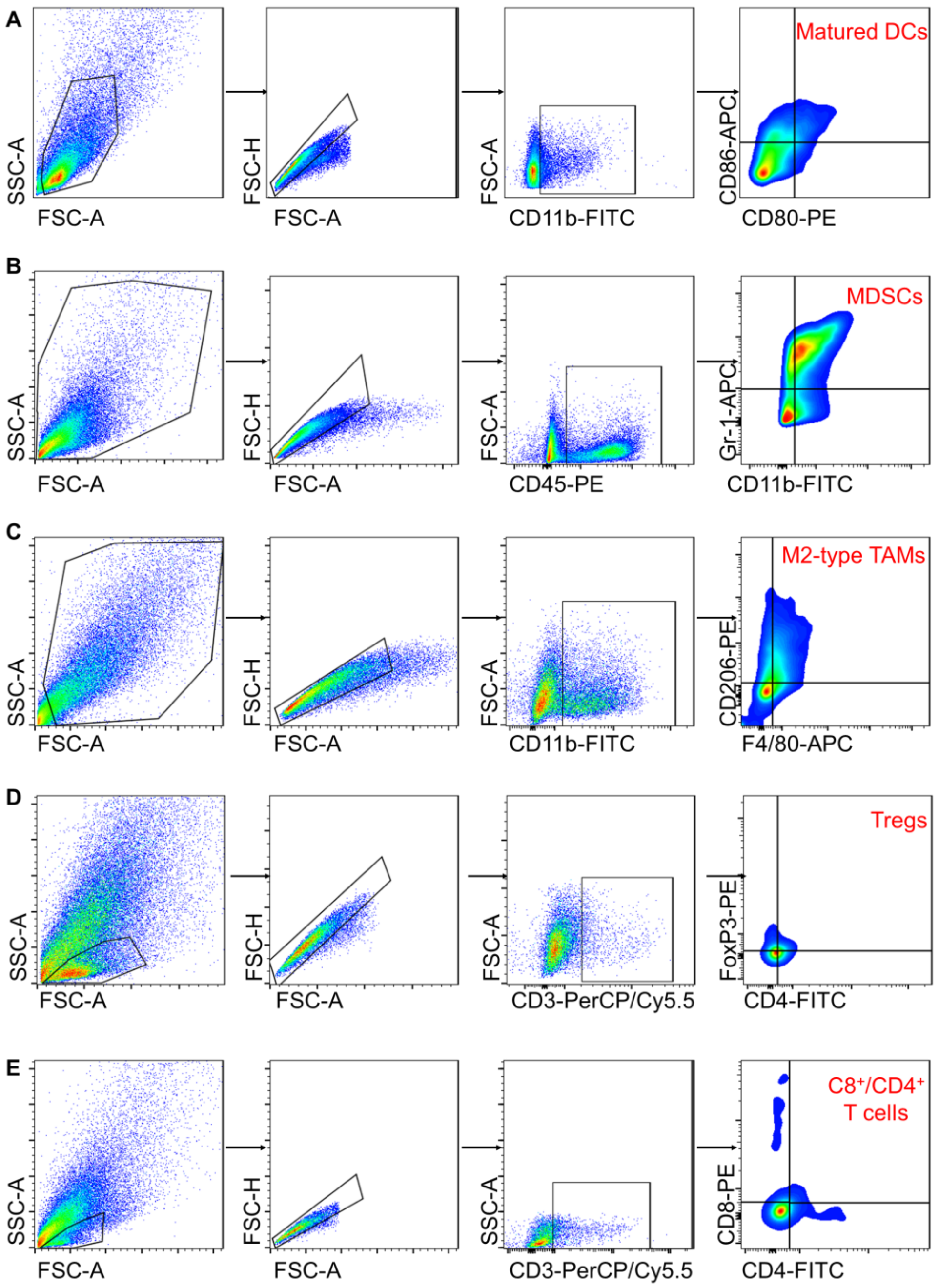

Fig. S13. The gating strategy of flow cytometry *in vivo* for determination of (A) DCs, (B) MDSCs, (C) M2-type TAMs, (D) Tregs, and (E) CTLs. For all panels we first created a cell gate based on SSC-A and FSC-A parameters. We continued by gating single cells by using the FSC-H and FSC-A parameters. Next, we distinguished each type of immune cells by their specific markers. Specifically, the gating method of DCs started from the selection of $CD11c^+$ cells from the single cell suspension of TDLNs, which were then selected by $CD80^+$ and $CD86^+$ for the analysis of

matured DCs. MDSCs were defined as $CD45^+CD11b^+Gr\text{-}1^+$. M2-type TAMs were defined as $CD11b^+F4/80^+CD206^+$. Tregs were separated by selection of $CD3^+$ cells from the single cell suspension of lymphocytes in tumors, which were then selected by $CD4^+$ and $FoxP3^+$. Effector T cells were further divided for $CD8^+$ and $CD4^+$ T cell populations based on the selection of $CD3^+$ cells from the single cell suspension of lymphocytes in tumors.

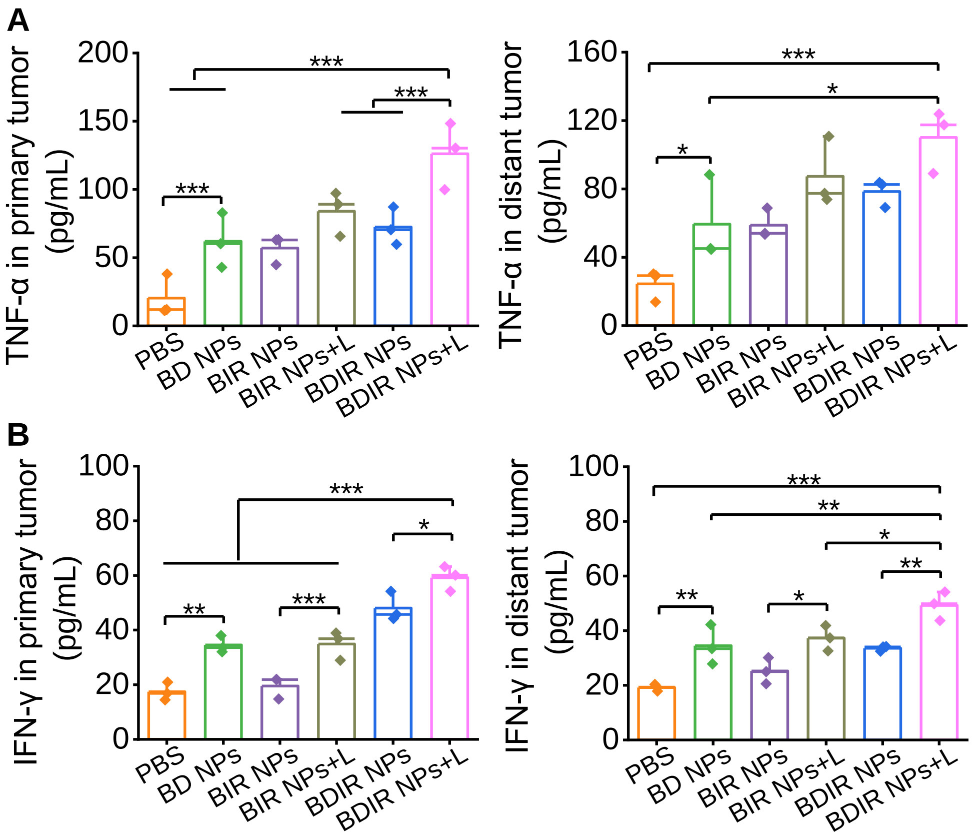

Fig. S14. The quantification of (A) TNF-α and (B) IFN-γ levels in bilateral 4T1 tumors after different treatments. Results are expressed as the mean ± SD. (n=3, statistical significance was analyzed using one-way ANOVA. *$p < 0.05$, **$p < 0.01$, ***$p < 0.001$).

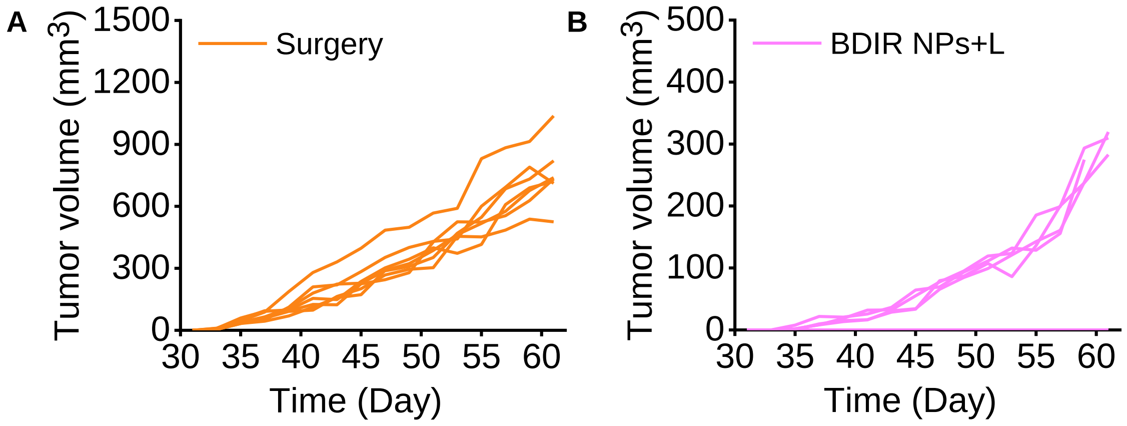

Fig. S15. Individual growth curves of rechallenged tumors for mice whose first tumors were treated with (A) surgery and (B) BDIR NPs+L (n=7).

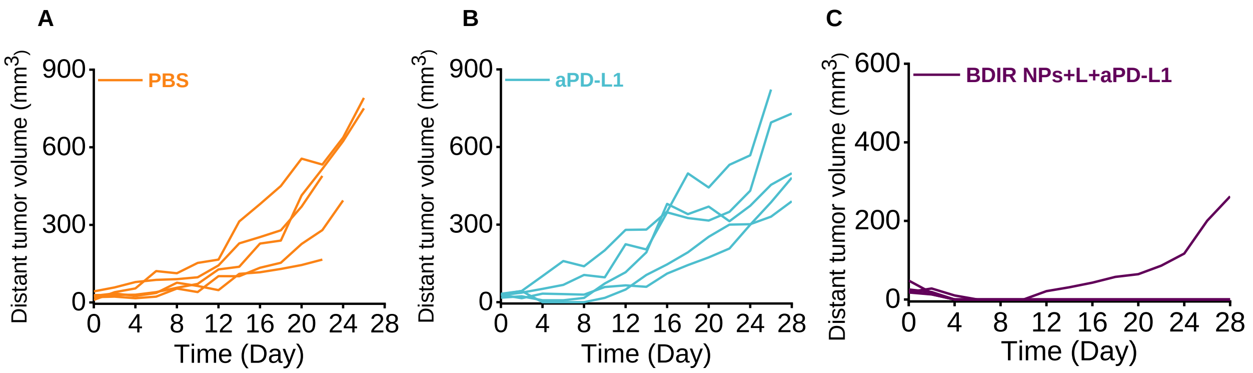

Fig. S16. *In vivo* anti-tumor effect of BDIR NPs combined with aPD-L1 treatment. The growth profiles of single distant 4T1 tumors subjected to (A) PBS, (B) aPD-L1, and (C) BDIR NPs+L+ aPD-L1 (n=5).

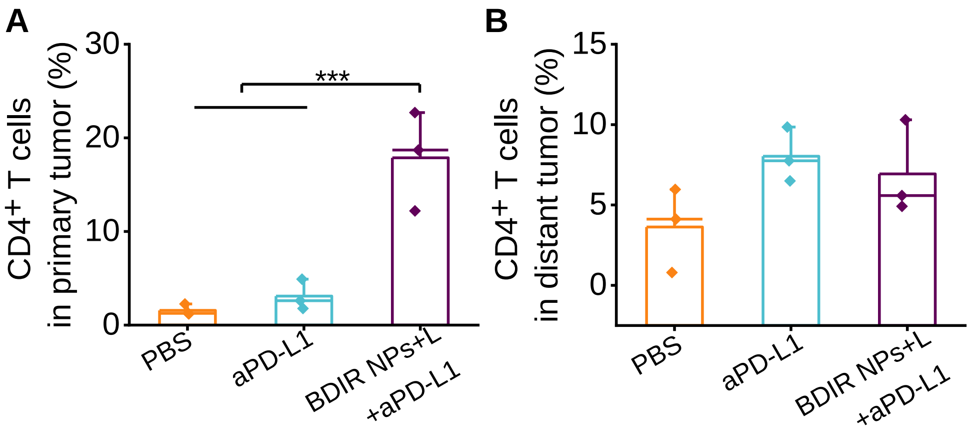

Fig. S17. $CD4^+$ T cell infiltration in (A) primary and (B) distant tumors after different treatments. Results are expressed as the mean ± SD. (n=3, statistical significance was analyzed using one-way ANOVA. ***$p < 0.001$).

Supplementary Tables

Table S1. Summary of NPs characterization (n=3).

**Entry**	**D_h (nm)**	**PDI**	**ZP (mV) (pH7.4)**	**TEM size (nm)**	**DL of IR780 (% w/w)**	**DL of DCF (% w/w)**	**EE of IR780 (%)**	**EE of DCF (%)**
BDIR NPs	121.6±5.4	0.157±0.012	-18.3±0.6	87.4±19.7	1.1±0.22	11.9±1.5	63.2±14.1	36.2±4.4
BIR NPs	97.6±6.3	0.388±0.105	-15.4±1.0	64.7±18.9	1.4±0.7	-	52.5±2.0	-
BD NPs	106.9±6.4	0.637±0.067	-14.4±2.2	62.9±13.4		8.1±1.2		21.7±2.2

Abbreviations: BDIR NPs, IR780 and DCF-coloaded BSA nanoparticles; BIR NPs, IR780-loaded BSA nanoparticles; BD NPs, diclofenac (DCF)-loaded BSA nanoparticles; D_h, hydrodynamic diameter; PDI, polydispersity index; ZP, zeta potential; DL, drug loading; EE, encapsulation efficiency

References

[1] Y. Zhai, M. Liu, T. Yang, J. Luo, C. Wei, J. Shen, X. Song, H. Ke, P. Sun, M. Guo, Y. Deng, H. Chen, Self-activated arsenic manganite nanohybrids for visible and synergistic thermo/immuno-arsenotherapy, J. Controlled Release 350 (2022) 761-776, https://doi.org/10.1016/j.jconrel.2022.08.054.
